# Impact of Electrolyzed Water on the Microbial Spoilage Profile of Piedmontese Steak Tartare

**DOI:** 10.1128/Spectrum.01751-21

**Published:** 2021-11-17

**Authors:** C. Botta, J. D. Coisson, I. Ferrocino, A. Colasanto, A. Pessione, L. Cocolin, M. Arlorio, K. Rantsiou

**Affiliations:** a Department of Agricultural, Forest and Food Sciences, University of Torino, Turin, Italy; b Dipartimento di Scienze del Farmaco, Università del Piemonte Orientale, Novara, Italy; c Laemmegroup S.r.l. a Tentamus Company, Moncalieri, Italy; University of Minnesota

**Keywords:** ground beef, electrolyzed water, spoilage microbiota, metataxonomy, biomarkers

## Abstract

A low initial contamination level of the meat surface is the *sine qua non* to extend the subsequent shelf life of ground beef for as long as possible. Therefore, the short- and long-term effects of a pregrinding treatment with electrolyzed water (EW) on the microbiological and physicochemical features of Piedmontese steak tartare were here assessed on site, by following two production runs through storage under vacuum packaging conditions at 4°C. The immersion of muscle meat in EW solution at 100 ppm of free active chlorine for 90 s produced an initial surface decontamination with no side effects or compositional modifications, except for an external color change that was subsequently masked by the grinding step. However, the initially measured decontamination was no longer detectable in ground beef, perhaps due to a quick recovery by bacteria during the grinding step from the transient oxidative stress induced by the EW. We observed different RNA-based metataxonomic profiles and metabolomic biomarkers (volatile organic compounds [VOCs], free amino acids [FAA], and biogenic amines [BA]) between production runs. Interestingly, the potentially active microbiota of the meat from each production run, investigated through operational taxonomic unit (OTU)-, oligotyping-, and amplicon sequence variant (ASV)-based bioinformatic pipelines, differed as soon as the early stages of storage, whereas microbial counts and biomarker dynamics were significantly distinguishable only after the expiration date. Higher diversity, richness, and abundance of Streptococcus organisms were identified as the main indicators of the faster spoilage observed in one of the two production runs, while Lactococcus piscium development was the main marker of shelf life end in both production runs.

**IMPORTANCE** Treatment with EW prior to grinding did not result in an effective intervention to prolong the shelf life of Piedmontese steak tartare. Our RNA-based approach clearly highlighted a microbiota that changed markedly between production runs but little during the first shelf life stages. Under these conditions, an early metataxonomic profiling might provide the best prediction of the microbiological fate of each batch of the product.

## INTRODUCTION

“*Battuta al Coltello di Fassona Piemontese*,” which literally means “meat of the Piedmontese cattle breed beaten (ground) with a knife,” is an Italian variant of the popular steak tartare: a raw beef dish freshly prepared in restaurants and immediately consumed with sauces and spices. In addition, its production as a ready-to-eat (RTE) food at the industrial level has increased considerably in recent years, and it has begun to populate the shelves of small- and large-scale retail trade outlets. Undoubtedly, consumption of raw ground beef improves the dietary intake of B vitamins, vitamin D3, phosphorus, zinc, and iron (1, 2), but it also raises strong concerns related to the intrinsic microbiological risks of this food, which are mainly represented by the potential presence of Listeria monocytogenes and enterohemorrhagic strains of Escherichia coli, such as Shiga toxin-producing E. coli (STEC) O157 (3–5). Besides being a favorable substrate for these and other pathogens, steak tartare is a very perishable product with a short shelf life that may span from 1 to a maximum of 2 weeks. Such perishability is mainly due to the absence of preservatives and thermal treatments, where the only hurdles available to minimize microbial proliferation are refrigeration, the gaseous composition of the packages, and the potential use of active packaging (6). However, it is noteworthy that nisin-coated packaging films that were successfully tested on beef cuts (7, 8) showed a limited effectiveness on ground beef (9). After all, the inner-contaminant microbiota of ground beef is not directly in contact with the antimicrobial bound to the external envelope of the active packaging.

The reduction of meat contamination before grinding seems therefore an alternative strategy to obtain shelf life prolongation (10–12). Undoubtedly, such a nonthermal decontamination strategy must be integrated in a context of maximum environmental hygiene to fully exert its effectiveness. Having in mind that all surfaces in contact with meat may represent sources of recontamination and cross-contamination between different batches (13, 14), attention to the applicability of electrolyzed water (EW) in meat decontamination has risen considerably in the last decade, in response to the industry’s demand for new effective and ecosustainable approaches. The efficacy of EW as a direct antimicrobial treatment has been intensively investigated and often proven during the last decade *in vitro* and, to a lesser extent, *in situ* on pork (15, 16), chicken carcasses (17, 18), cattle carcasses (19, 20), and beef cuts (21–25). In the majority of these experiments, neutral electrolyzed water and slightly acidic electrolyzed water produced from single-chamber generators have been used because of their neutral or nearly neutral pH values (26), while the chosen modes of use have substantially been the spraying or immersion of the meat. Notably, these two approaches can lead to different results in term of decontamination effectiveness, and usually, higher concentrations of free chlorine are used in the spraying methods to achieve satisfactory results (20, 22, 27).

Regardless of the mode of use and type of meat treated, the main concern in using a chlorine-based sanitizer like EW directly on a foodstuff is the potential increase in the chlorate content in the final product (24), which has led to two diametrically opposite positions in the European Union and U.S. food market regulations. In Europe, the only nonthermal treatment allowed so far on fresh meat is lactic acid (28), while in the United States, chlorinated water is approved and widely used in the poultry slaughtering chain (29). The discoloring and the oxidation of lipidic fractions might also represent other potential hurdles for the application of EW in meat industries, although until now, pilot-scale trials have highlighted negligible effects on such features (16, 30). Always taking into account effectiveness and side effects, applied research has mostly pointed out the instant antimicrobial effect of EW upon treatment of meat, while minor attention has been paid to its long-term impact on microbiota evolution during the shelf life (25, 31).

Here, we aim to define the feasibility of a pregrinding decontamination performed by immerging meat trimmings in EW at 100 ppm of free chlorine for 90 s in the real context of a steak tartare processing line. The effectiveness of EW and its impact on the shelf life were monitored in two distinct production runs. The microbiological, metataxonomic, and chemical profiles were comprehensively analyzed in order to highlight in parallel the impact of the antimicrobial treatment and the autochthonous/allochthonous microbiota of each production run on the microbiological fate of Piedmontese steak tartare.

## RESULTS

### Short- and long-term impacts of the initial EW treatment.

In a preliminary experiment, we observed a maximum reduction of total viable counts (TVC) on meat trimming surfaces after 90 s of dipping in EW at 100 ppm of free chlorine content (FCC), while treatment with lower FCC levels (25 and 50 ppm) was ineffective for the same time frame (Fig. S1 in the supplemental material). This EW treatment was applied in two industrial production runs of Piedmontese steak tartare, each constituted by two replicates, by immerging the meat trimmings before grinding. Treated and control lots of ground beef were then followed during vacuum package storage at 4°C for 21 days (Fig. S2).

**FIG 1:**
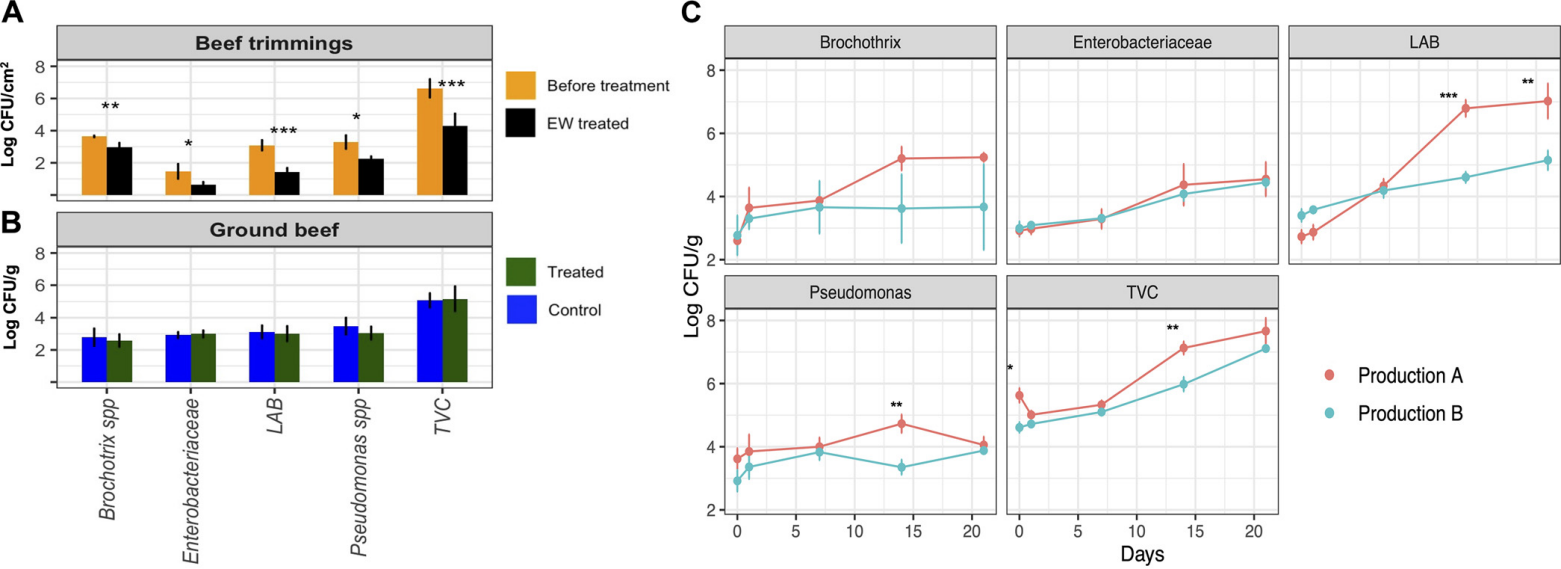
Microbiological impact of EW and spoilage dynamics. Bar plots and graphs of dynamics showing the viable counts (mean ± SD) of total bacteria (TVC, total viable count) and four spoilage bacterium populations (LAB, lactic acid bacteria). (A) Superficial viable counts (Log CFU/cm^2^) of meat trimmings before treatment (sampled before splitting the batch into treated and control lots; 10 cm^2^, *n* = 3 in each sampling) are compared to part of the same trimmings that underwent EW immersion treatment (10 cm^2^, *n* = 3 in each sampling). (B) Viable counts (Log CFU/g) of treated ground beef are compared to those of the control lot. (C) Spoilage dynamics of ground beef during the 21 days of vacuum storage; data from each sampling point are grouped by production run. Asterisks highlight significant differences (Student’s *t* test; *, *P* < 0.05; **, *P* < 0.01; ***, *P* < 0.001).

The treatment confirmed *in situ* its ability to significantly (*P < *0.05) reduce the TVC on meat surfaces, with an average decrease of 1 Log CFU/cm^2^, as well as to reduce the *Brochothrix*, Pseudomonas, lactic acid bacterium (LAB), and *Enterobacteriaceae* populations (Fig. 1A). The dipping procedure of the trimmed meat resulted in a decrease of the initial FCC of EW (from 100 ± 2 ppm [mean ± standard deviation] to 3 ± 1 ppm); meanwhile, at the end of the treatment, the chlorate content of the meat was under the detection limit (0.01 mg/kg) while its color changed visibly from red to gray (Fig. S3). However, after the grinding process, the treated and untreated meat samples showed the same count levels and external color (Fig. 1B and Fig. S3). It is noteworthy that the TVC of the meat grinder and stainless-steel basket used for the EW treatment showed significantly (*P < *0.05) lower microbial loads than were detected on meat surfaces, with average counts of 2.97 ± 1.12 and 2.04 ± 1.52 Log CFU/cm^2^ on the meat grinder and basket, respectively.

The microbial counts during the shelf life differed in relation to the two production runs (A and B) rather than the two lots (EW and untreated control); therefore, at each sampling point of production runs A and B, the treated and control ground beef were grouped together. The microbiological dynamics showed a time course increase of TVC and LAB in both production runs, with significantly higher counts for these two populations in production run A starting from the 14th day (Fig. 1C).

Aside from the metabolites potentially related to the impact of EW and the microbiological metabolism, the peroxide contents were also quantified, with significant differences not observed between the EW treatment and control lots but only between the two production runs (Table S3). As far as degradation of the myofibrillar protein fractions, except for a slight proteolysis observed for troponin T after the 14th day, we could not detect differences associated with the EW treatment or the two production runs (data not shown). Finally, the data related to proximate composition and the fatty acid methyl esters (FAMEs) in the lipidic fraction confirmed the differences between the meat from the two production runs (A and B), as evidenced by the principal-component analysis (PCA) applied to these data (Fig. S4).

### Dynamics of spoilage metabolomic biomarkers.

A total of 11 volatile organic compounds (VOCs), selected taking into account a previous work on meat treated with EW (25), were quantified in the headspace of vacuum-packaged steak tartare and showed increasing concentrations during the storage period. Ethyl-hexanoate, hexanal, and 1-hexanol represented the most abundant compounds (Table S1). No significant differences in VOC concentrations were observed between the two treatments or between the two production runs.

**FIG 2:**
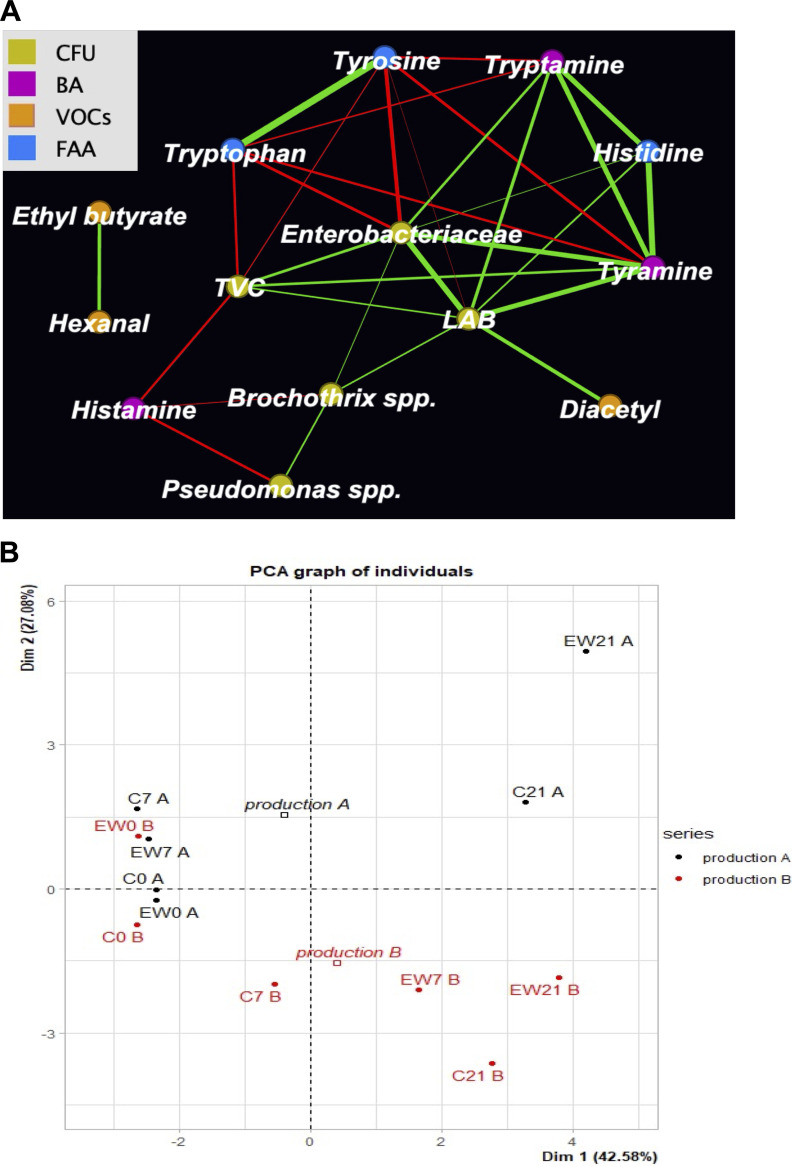
Presence of spoilage biomarkers and their correlations with microbial populations. (A) Network displaying all significant Spearman’s correlations (*P* [FDR] < 0.001) existing among viable counts, free amino acids (FAA), biogenic amines (BA), and volatile organic compounds (VOCs). For colors of nodes, refer to the key. Colors of the edges indicate positive (green, Rho > 0.5) and negative (red, Rho < −0.5) correlations, and thickness is proportional to the absolute Rho value. Node dimension and edge lengths have no specific meaning. (B) PCA obtained by using VOC, FAA, and BA data. PCA score scatterplots of data for meat samples (C, control; EW, treated). The PCA model was obtained with the first two principal components, which explained 69 and 66% of the total response variance.

Except for histidine, all free amino acid (FAA) concentrations decreased significantly during the shelf life and differed at each sampling point between production runs A and B but not in relation to the EW treatment with 100 ppm of FCC (Table S2). As for biogenic amines (BAs), we observed after the 7th day of storage an increasing accumulation of tyramine and, to a lesser extent, of tryptamine. In contrast, tyrosine and tryptophan decreased progressively after the 7th day, and their concentrations were therefore negatively (false discovery rate-adjusted *P* value {*P* [FDR]} of <0.001; Rho < 0.5) correlated with the derived biogenic amines. Interestingly, this expectable inverse correlation between an amino acid and its derived biogenic amine was not observed for histidine and histamine, with no changes of histidine concentration during the storage time. Finally, 2-phenylethylamine was not detected in the samples. Extending the correlation analysis to microbiological dynamics and chemical compounds (VOCs, FAAs, and BAs), we observed an overall positive correlation (*P* [FDR] < 0.001; Rho > 0.5) between LAB/*Enterobacteriaceae* counts and the presence of tryptamine and tyramine. Notably, only the LAB counts were positively correlated (*P* [FDR] < 0.001; Rho > 0.5) with diacetyl, regardless of the production run or treatment lot (Fig. 2A).

A multivariate PCA of the VOC, FAA, and BA data was performed, and the results are presented in Fig. 2B. Even with these parameters, a separation of the two series along the principal-component 2 (PC2) axis is evident, while the storage time affects the position of the samples along PC1.

### Microbiota composition of steak tartare.

**FIG 3:**
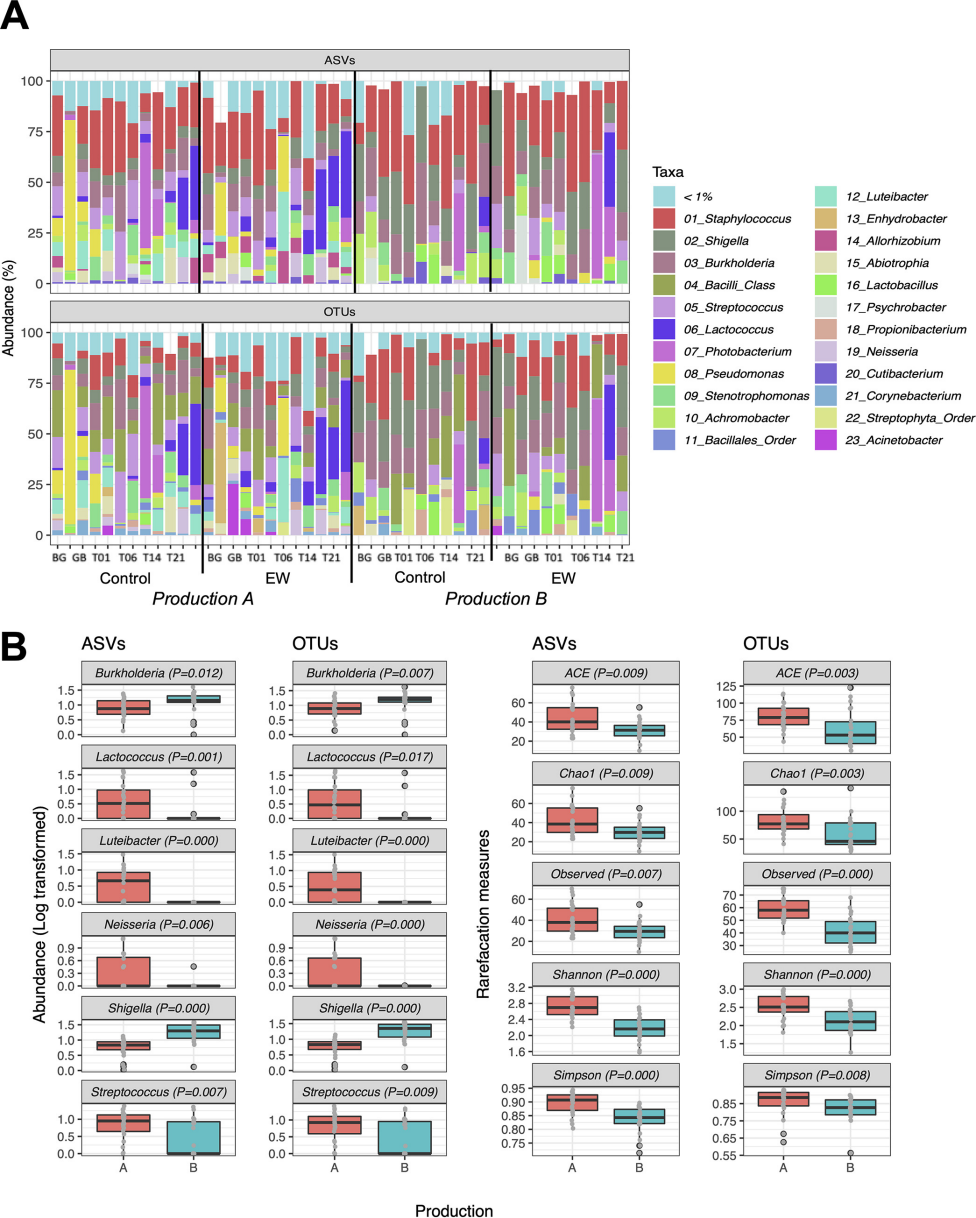
Compositional microbiota of steak tartare. (A) Taxonomic rank levels achieved by ASV- and OTU-based approaches, expressed as percentages of sequences assigned to a given taxonomic rank compared to the total number of sequences. Stacked bar plots show microbiota compositions (relative abundances of ASVs and OTUs in percentages) at the genus level, as shown in the color key; samples are sequentially grouped according to the sampling points (BG, before grinding; GB, ground beef; and T, storage time [days]), lots (control and EW), and production runs (A and B). (B) Box plots of Log-transformed abundances (ASVs and OTUs) of the core genera in the two production runs; *P* value of Wilcoxon’s test [FDR adjusted] is displayed.

Following the microbiological and chemical analyses, we explored the bacterial biodiversity of 46 samples collected during the product shelf life by the parallel use of operational taxonomic units (OTUs) obtained through *de novo* clustering at 97% and amplicon sequence variants (ASVs) (Fig. 3). Totals of 2,377,251 and 2,516,054 sequences were obtained from the ASV- and OTU-based pipelines, respectively. The sequences were aligned at 97 and 99% similarity to reference databases for OTUs and ASVs, respectively; 60% of the ASVs reached the species level, while only 40% of the OTUs were assigned to a defined species. In relation to this difference in the taxonomic resolution, we initially explored the microbiota composition at the genus level, or the next level up if genus was not reached. In this frame, the genera *Shigella*, Staphylococcus, and *Burkholderia* were ubiquitous and represented more than 10% of total average abundance in both the ASV- and OTU-based taxonomies (Fig. 3A). The microbiota of steak tartare also encompassed Streptococcus, *Stenotrophomonas*, *Achromobacter*, *Lactococcus*, Pseudomonas, *Photobacterium*, *Luteibacter*, *Lactobacillus*, *Abiotrophia*, and *Neisseria*, which were all found in more than 10 samples. In relation to the approach used, we observed a higher presence of Staphylococcus in the ecology based on ASVs (Wilcoxon’s test, *P* [FDR] < 0.001) and a peculiar presence of *Propionibacterium* and *Bacilli*/*Bacillales* in the OTU-clustering approach. This different taxonomic assignment was minimally dependent on the database used and mostly related to the pipeline, as demonstrated by aligning ASVs on the 97% database used for OTU taxonomy (Table S4).

Permutational analysis of variance (PERMANOVA) confirmed that the greatest portion of compositional variability was determined by the two different production runs considered, in both ASV-based (*R*^2^ = 0.106; *P* < 0.001) and OTU-based (*R^2^* = 0.130; *P < *0.001) microbiota, while the EW treatment and storage time did not determine marked changes of bacterial communities (Table 1). In particular, LAB of the genera *Lactococcus-*Streptococcus and Gram-negative genera *Luteibacter-Neisseria* were significantly more abundant in production run A, which also showed higher richness and diversity (Fig. 3B). On the other hand, we observed significantly greater abundances of *Shigella* and *Burkholderia* in production run B.

**TABLE 1:** Variance in biological dissimilarity among bacterial communities explained by each categorical variable (production run, time, and lot) or their interactions^*a*^

Explanatory variable(s)^*b*^	ASVs		OTUs	
	*R* ^2^	*P* value^*c*^	*R* ^2^	*P* value
Single variable				
Production run (A, B)	0.106	0.001*	0.130	0.001*
Time (BG, GB, T0, T01, T06, T14, T21)	0.097	0.434	0.095	0.362
Treatment lot (control, EW)	0.045	0.009	0.053	0.005
Interaction				
Treatment lot × production run	0.028	0.111	0.025	0.152
Treatment lot × time	0.092	0.594	0.099	0.284
Production run × time	0.087	0.255	0.103	0.054
Treatment lot × production run × time	0.081	0.375	0.062	0.696

a Variance explained (*R*^2^) and statistical significance (*P* value) quantified by permutational analysis of variance (PERMANOVA) test of Bray-Curtis dissimilarity.

b BG, before grinding; GB, ground beef; T0, T01, T06, T14, T21, storage times (days); EW, electrolyzed water treatment.

c Since *P* values result from a 999-permutation test, they are only reported as significant (*) down to 0.001.

No significant variations of alpha-diversity parameters or relative abundances were observed before and after the EW treatment or between meat trimmings and ground beef in either the control or the treated lot.

As for the inferred metagenome functions, pathway enrichment analysis showed that 23 metabolic pathways were differentially represented in the two production runs (GAGE [generally applicable gene set enrichment] statistics, *P < *0.001). In particular, three predicted KEGG pathways for carbohydrate metabolism (map00562, map00040, and map00500) and three for amino acid metabolism (map00270, map00260, and map00280) were significantly overrepresented in production run A. Moreover, lipopolysaccharide biosynthesis was more highly represented in production run B, while pathways responsible for peptidoglycan and glycan biosynthesis were more abundant in production run A (data not shown).

### Microdiversity within bacterial communities and links with spoilage biomarkers.

To overcome the resolution limits of the OTU approach, the within-genus microdiversity of the most abundant OTUs (>1% of average abundance) was investigated at single-nucleotide-based resolution by oligotyping 1,791,996 paired-end reads from the genera Staphylococcus, *Burkholderia*, *Shigella*, Streptococcus, *Lactococcus*, *Stenotrophomonas*, *Photobacterium*, Pseudomonas, and *Luteibacter*, the class *Bacilli*, and the order *Bacillales*. A total of 181 oligotypes belonging to 29 different species were detected, with nine genera identified that encompassed a maximum of nine species (Pseudomonas) and a minimum of one species (*Paraburkholderia*, *Stenotrophomonas*, and *Luteibacter*). All oligotypes from *Burkholderia* OTUs were identified as Paraburkholderia fungorum, whereas sequences included in the *Bacillales* and *Bacilli* OTUs were assigned to different Staphylococcus species (Table 2). For the genus *Shigella* and several Staphylococcus oligotypes, it was not possible to reach an unequivocal identification at the species level, but by investigating their distribution in the set of samples through a SparCC (sparse correlations for compositional data) cooccurrence analysis, we noticed in the resulting network graph a homogeneous clustering of their oligotypes (Fig. S5). Overall, the cooccurrence network highlighted species-specific cooccurrence patterns in most of the oligotyped genera: i.e., oligotypes tended to group in modules as a function of the species to which they belonged. This is particularly evident for oligotypes assigned to Lactococcus piscium, Luteibacter rhizovicinus, Paraburkholderia fungorum, Staphylococcus sciuri, Streptococcus mitis, and Streptococcus sanguinis and, indeed, for two undefined taxa belonging to the *Shigella* and Staphylococcus genera. Since oligotypes of the same taxa distributed in the same samples likely indicate the presence of a homogeneous ecotype, the oligotype frequencies were cumulated in each species-specific module by considering them as single biological entities in the following analysis. Overall, all species were present in both production runs, except for Lactococcus lactis oligotypes, which were found only in production run A.

**TABLE 2:** Numbers of oligotypes and the species they belong to present in the selected core OTUs^*a*^

OTU identity (no. of oligotypes)	No. of sequences	No. of taxa	Oligotype identity (no.)	No. of sequences	Cooccurrence module or ungrouped oligotypes (no.)	% of sequences represented
*Lactococcus* (14)	120,311	2	Lactococcus lactis (8)	18,415	M5 (1)	53.1
					Ungrouped (7)	46.9
			Lactococcus piscium (6)	101,896	M4 (6)	100.0
*Luteibacter* (10)	57,229	1	Luteibacter rhizovicinus (10)	57,229	M5 (8)	96.1
					Ungrouped (2)	3.9
*Burkholderia* (26)	241,380	1	Paraburkholderia fungorum (26)	241,380	M1 (23)	99.9
					Ungrouped (3)	0.1
*Photobacterium* (20)	132,246	3	*Photobacterium carnosum* (6)	57,529	M7 (2)	31.7
					Ungrouped (4)	68.3
			Photobacterium iliopiscarium (9)	32,702	M7 (5)	14.9
					Ungrouped (4)	85.1
			Photobacterium phosphoreum (5)	42,015	M7 (5)	100.0
Pseudomonas (16)	72,770	9	Pseudomonas asturiensis (1)	83	Ungrouped (1)	100.0
			Pseudomonas caeni (1)	1,094	Ungrouped (1)	100.0
			Pseudomonas chengduensis (1)	93	Ungrouped (1)	100.0
			Pseudomonas fragi (1)	579	M9 (1)	100.0
			Pseudomonas migulae (1)	819	Ungrouped (1)	100.0
			Pseudomonas paralactis (1)	1,666	Ungrouped (1)	100.0
			Pseudomonas psychrophila (2)	7,808	M9 (1)	42.3
					Ungrouped (1)	57.7
			Pseudomonas turukhanskensis (5)	27,223	M10 (4)	99.4
					Ungrouped (1)	0.6
			Pseudomonas veronii (3)	33,405	M8 (3)	100.0
*Shigella* (18)	299,714	1	Shigella sonnei*/flexneri* (18)	299,714	M11 (18)	100.0
*Bacillales* (12)	57,933	3	Staphylococcus cohnii (1)	163	Ungrouped (1)	100.0
			Staphylococcus saprophyticus (10)	56,667	M2 (1)	0.2
					Ungrouped (9)	99.8
			Staphylococcus xylosus (1)	1,103	Ungrouped (1)	100.0
*Bacilli* (8)	312,322	2	Staphylococcus aureus*/simiae* (7)	311,291	M0 (1)	0.1
					Ungrouped (6)	99.9
			Staphylococcus capitis*/caprae* (1)	1,031	Ungrouped (1)	100.0
Staphylococcus (20)	277,571	5	Staphylococcus aureus*/simiae* (2)	12,380	M0 (1)	97.7
					Ungrouped (1)	2.3
			Staphylococcus cohnii (1)	882	M2 (1)	100.0
			Staphylococcus saprophyticus (1)	10,998	Ungrouped (1)	100.0
			Staphylococcus sciuri (15)	252,708	M12 (14)	99.9
					Ungrouped (1)	0.1
			Staphylococcus warneri (1)	603	Ungrouped (1)	100.0
*Stenotrophomonas* (18)	88,541	1	Stenotrophomonas pavanii (18)	88,541	M13 (8)	48.9
					M14 (4)	30.3
					M15 (4)	20.6
					Ungrouped (2)	0.2
Streptococcus (19)	131,979	4	Streptococcus cristatus (1)	528	M17 (1)	100.0
			Streptococcus mitis (10)	93,558	M16 (8)	98.3
					Ungrouped (2)	1.7
			Streptococcus salivarius (1)	1,473	Ungrouped (1)	100.0
			Streptococcus sanguinis (7)	36,420	M17 (7)	100.0

a Core OTUs were those with >1% average abundance; OTUs were determined to the genus level or the next higher level. Highly cooccurring oligotypes were detected by analyzing the SparCC network topology (Fig. 4) through the algorithm described in reference 84 and grouped in modules (Fig. S3).

Noteworthy at the species level was the similarity between the relative abundance profiles produced by the oligotyped OTUs and ASVs. Despite the pipelines used to generate the sequences and the alignments performed on different databases, we observed similar sets of predominant species, namely, Lactococcus piscium, Staphylococcus sciuri, Photobacterium phosphoreum, and Luteibacter rhizovicinus. For other taxa, such as Streptococcus and *Stenotrophomonas*, the species assignments were more discordant (Fig. S6).

*Lactococcus* and *Photobacterium* were significantly, if not exclusively present after the end of the product shelf life, regardless of the approach used (Fig. 4). Noticeably, the OTU-based approach could not discriminate at the species level within the genus *Lactococcus*, while ASVs and oligotyping highlighted the specific presence of Lactococcus piscium only from the 14th day of storage and regardless of the production run of origin.

**FIG 4:**
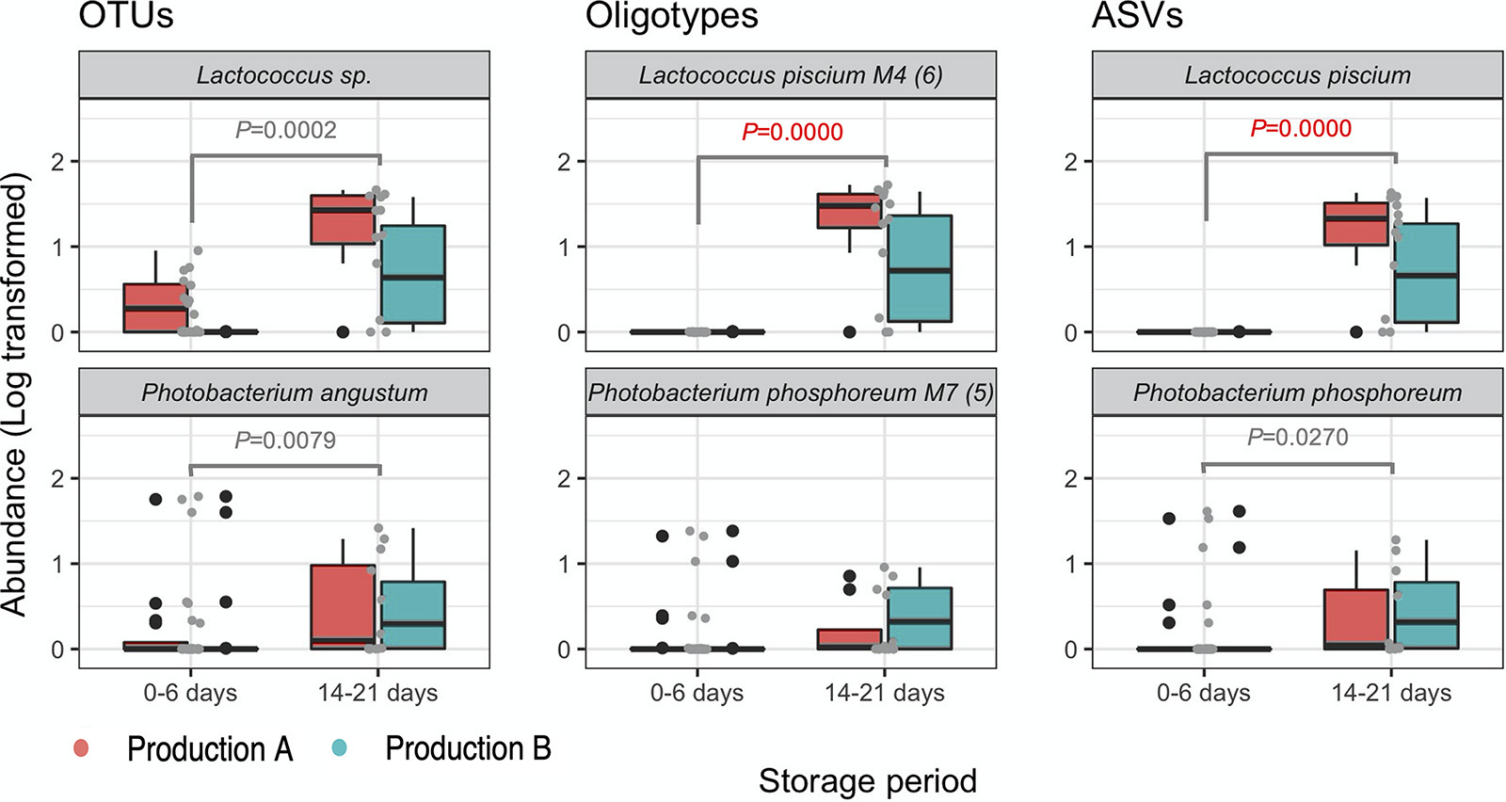
Distribution of OTUs, oligotypes, and ASVs assigned to *Lactococcus* and *Photobacterium* species in the first week of shelf life and in the expired product. Log-transformed abundances of ASVs, OTUs and oligotypes (grouped in each species by cooccurring modules) are shown. The *P* values of Wilcoxon’s test [FDR adjusted] are displayed and highlight significant differences between days 0 and 6 and days 14 to 21, regardless of the production run of origin, which are displayed with red (production run A) and cyan (production run B) box plots.

**FIG 5:**
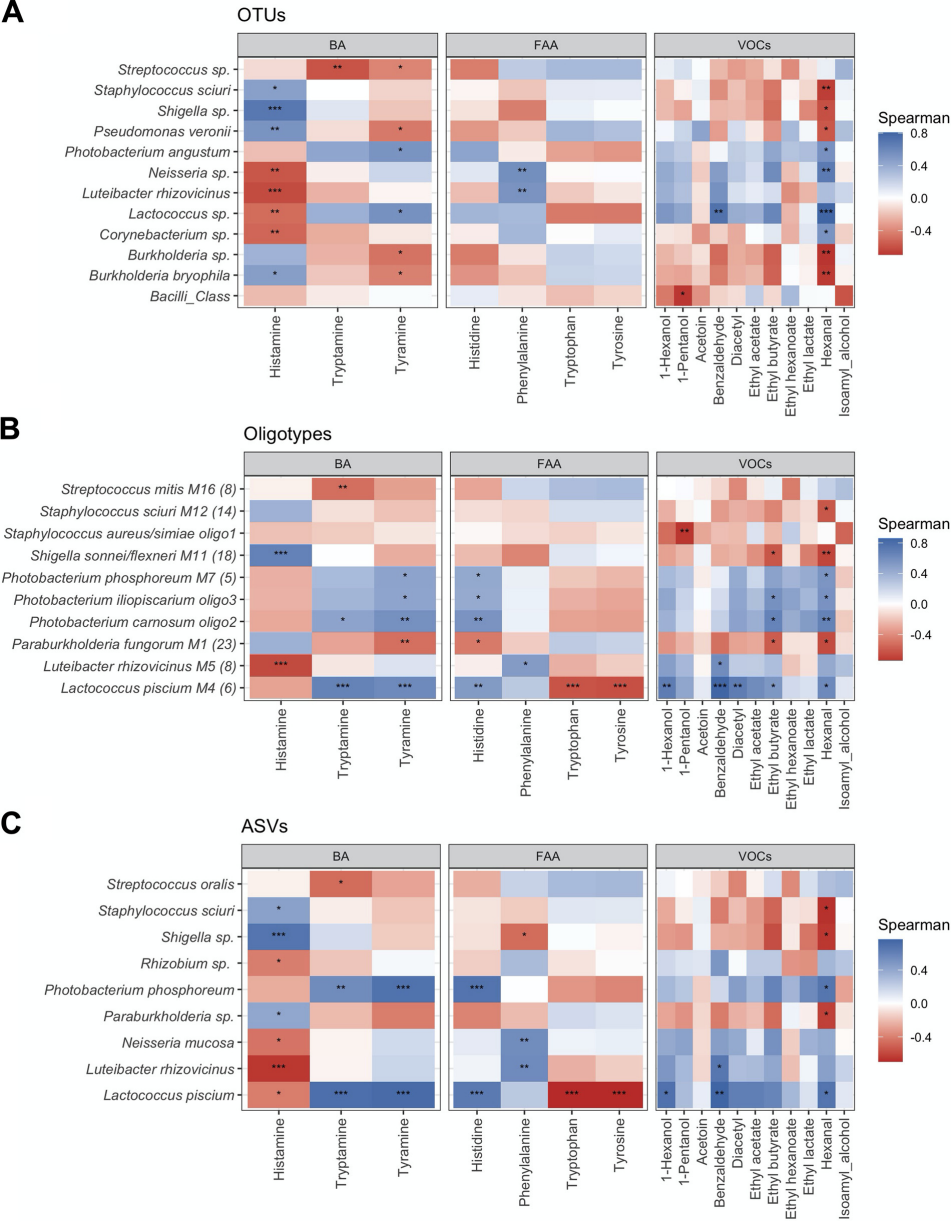
Correlation between metataxonomic and chemical data. Tile plots showing existing correlation between chemical data (FAA, free amino acids; BA, biogenic amines; VOCs, volatile organic compounds) and OTU, oligotype, and ASV abundances. Colors represent levels of Spearman’s Rho correlation from −1 to 1 as shown in the key, and significant positive and negative correlations are highlighted with asterisks (FDR-adjusted *P* values: *, *P* < 0.05; **, *P* < 0.01; ***, *P* < 0.001).

Regarding links among bacteria and the metabolites potentially produced by their anabolic and catabolic activities, we investigated the existing correlations between FAA, BA, and VOC concentrations and the compositional microbiota of steak tartare at the highest taxonomic ranks achievable, respectively by OTUs, oligotypes, and ASVs (Fig. 5). Regardless of the approach used, the relative abundances of *Shigella* species and Luteibacter rhizovicinus were positively and negatively correlated (*P* [FDR] < 0.001), respectively, with the presence of histamine. On the other hand, only comparing oligotype and ASV abundances with FAA and BA abundances, we could appreciate a significant correlation (*P* [FDR] < 0.001) between Lactococcus piscium and the reduction of tryptophan/tyrosine, as well as the subsequent accumulation of tryptamine/tyramine. Tyramine accumulation was positively correlated with Photobacterium phosphoreum ASVs also and, to a lesser extent, with all oligotypes identified in the *Photobacterium* genus (*P* [FDR] < 0.05). In accordance with the high concentrations of hexanal shown in the late stages of storage, this aldehyde showed the highest number of positive correlations with dominant taxa in the final storage period. Taking into account the aforementioned correlation between diacetyl and LAB viable counts, it is remarkable that Lactococcus piscium oligotypes were positively correlated (*P* [FDR] < 0.01) with the accumulation of this ketone in the steak tartare.

## DISCUSSION

This study aimed to provide a comprehensive scenario of the impact exerted by a pregrinding treatment with EW on two different production runs of Piedmontese steak tartare. For this purpose, the microbiota evolution was monitored during the shelf life in vacuum packaging by using RNA-based metataxonomic analysis at the highest resolution achievable and was matched to the spoilage biomarkers detected.

The antimicrobial activity of this treatment on meat trimmings has been preliminarily proven *in vitro* and then confirmed *in situ* by an overall decrease of spoilage bacteria on meat surfaces. Unlike our previous survey, in which the same EW treatment applied by spraying did not modify the beef color (25), here, the immersion of trimmings caused a color modification that would discourage the use of such an approach when the meat processing does not include a subsequent manipulation (grinding, cooking, or mixing with ingredients) that could likely mask this side effect (32). With regard to other potential side effects, the treatment did not increase peroxide formation or degradation of the protein structure, confirming the positive outcomes previously observed in chicken breasts and frozen beef treated with different types of EW (15, 24, 30, 33). The main safety risk in using chlorinated water on foods was excluded too, since chlorate residues were not detectable and, thus, largely below the limit imposed by the European Food Safety Authority (34).

In spite of this significant but slight decontamination prior to grinding, the evenness of the counts between treated and untreated ground beef highlighted the poor suitability of this approach for overall improvement of the microbiological quality of steak tartare and extension of its shelf life. Meat grinders are a well-recognized source of contamination and biofilms (35–37); however, the low superficial counts on the grinder used here before the production runs and the unaltered microbiota composition of the meat after grinding lead us to exclude the direct contribution of this processing step in the microbiological levelling between treatment lots. Moreover, these results confirm the minimal or momentary impact of the grinding step in the final ground beef microbiota composition and counts (38).

As far as the direct bactericidal effect of EW is concerned, chlorine-based disinfectants have been proven to promote in several bacteria the viable but nonculturable (VBNC) state, a stress condition that determines a reduced cultivability and may lead to overestimation of the immediate impact of any disinfection treatment in terms of microbiological counts (39–42). Moreover, a recent metadata analysis highlighted the lower decontamination capability of EW treatments on muscle meat in comparison with their effects on drier and more-smooth-surfaced foods (eggs and vegetables), regardless of the FCC, time, and temperature of the treatments performed (43). A lower effectiveness is likely due to the higher protein content of meat exudates, which can act as a protective layer and limit the direct oxidation of surface microbiota (27). In this frame, induction to VBNC status might have caused the transient effectiveness observed on meat trimmings, since the resuscitation from this metabolic condition can take place in the treated food when optimal conditions are quickly restored (44, 45). Indeed, bacterial translocation from treated meat surfaces to ground beef can offer a greater availability of space and nutrients, together with major protection from EW-induced oxidation. It is important to underline that our results do not exclude *a priori* the potential efficacy of this and other similar oxidizing treatments toward specific pathogens in highly contaminated or experimentally inoculated meats (10, 46). Nevertheless, targeted pathogen inhibition and VBNC induction are aspects beyond the scope of this untargeted observational study of the potentially active microbiota.

Likely due to the transitory, short-term effect, the spoilage dynamics of the steak tartare were not significantly impacted by the initial EW immersion. The composition of the potentially active microbiota differed significantly between the two production runs from the first day, and minimal longitudinal changes were observed during the time course. In parallel, viable counts and metabolite dynamics were distinct between the production runs, but only after the first week of vacuum storage could we observe an acceleration of spoilage dynamics in production run A, both in terms of LAB growth and metabolite production. The high batch-to-batch microbiota diversity is not surprising for minimally processed food like ground beef (9, 25, 38), since it is strongly related to different contaminations occurring on carcasses or primal cuts during the slaughtering and dissection stages (47).

More intriguing is the limited temporal succession of taxa observed during the first week of storage. Such temporal succession has largely been described in vacuum-packaged meat and is characterized by the gradual shift from a highly diversified microbiota with several aerobic and Gram-negative organisms toward a less miscellaneous ecology dominated by a few Gram-positive organisms, mainly lactic acid bacteria (47–49). Reduction of biodiversity and the establishment of an oligarchic microbiota are clear starting signals of spoilage phenomena and form a prelude to the subsequent increase of viable counts and perceptible organoleptic alterations (38, 50, 51). These considerations, together with the microbiological counts and chemical data, highlighted that both production runs did not spoil within the shelf life and were substantially acceptable beyond its end as well; for instance, among the VOCs, only ethyl-hexanoate was over the threshold of sensory perception on the 20th day (52).

However, the increase in Lactococcus piscium abundance at the end of the shelf life, as well as its correlation with catabolism of two amino acids and diacetyl production, pointed out an initiating spoilage phase led by this species. This psychrotrophic LAB is the only member of the genus *Lactococcus* with recognized spoilage activity, exerted in the late phases of cold storage by dominating the microbiota of vacuum-packaged meats (53–55). In particular, it metabolizes acetoin and diacetyl from amino acids and glycerol when glucose has already been consumed by other LAB species with faster-growing phenotypes (51, 56). In relation to this slow-growing spoilage strategy, its limited detectability in plate counts with incubation over 30°C, and its presence in both production runs, the development of Lactococcus piscium was not a determinant of the different microbiological dynamics of spoilage populations observed in production runs A and B. In particular, microbiological counts highlighted a faster spoilage dynamic in production run A. Photobacterium phosphoreum was another endpoint indicator of ground beef shelf life in both production runs. Its increasing presence was observed in the late storage phases and was strongly correlated with biogenic amine production, in agreement with the outcomes of targeted investigations of this bioluminescent bacterium (57, 58). Therefore, the distinctive signals of faster spoilage in production run A were present from the earliest shelf life stages, including the greater microbial complexity, the higher presence of *Firmicutes*, particularly Streptococcus, and the overrepresentation of inferred pathways of carbohydrate metabolism (12, 38).

While taking into account its dimensional limitations, our applied investigation suggests the relevance of production run-by-production run metataxonomic profiling as a strategy to foresee the maximal storage period of each product batch, an untargeted microbiota profiling that has to be performed at the highest taxonomic resolution to be fully informative for microbial habitats like foodstuffs, in which a few phylogenetically close and recurring species are responsible for the spoilage dynamics (59). In this frame, the parallel use of three bioinformatic pipelines has confirmed all taxonomic-rank resolution limits of the traditional OTU clustering approach in comparison with paired-end assembling of ASVs (60, 61). Indeed, although the OTU and ASV approaches showed a high degree of agreement in terms of the main taxon composition and alpha diversity metrics (62, 63), going deeply into the microdiversity, a lack of resolution was observed for the OTU approach, regardless of the final database used, and it was only partially improved by the subsequent oligotyping.

Undoubtedly, our untargeted survey also showed limits of resolution as a whole, mainly in terms of the detection of pathogens and their certain identification. For instance, we could not depict the microdiversity within the *Shigella* genus, which can potentially harbor pathogenic species, and this genus was highly abundant in all ground beef samples. This is not surprising, since it is even difficult to discriminate between *Shigella* and Escherichia at the genus level if only single genes are used as taxonomic keys (64, 65). However, the oligotype cooccurrence analysis has highlighted in this product an Escherichia*/Shigella* population homogeneously distributed in all samples that likely encompasses a unique ecotype/species not identifiable on the basis of the 16S gene (13, 66, 67). As far as the presence of other pathogens typically present in ground beef, we did not detect *Listeria* spp., regardless of the bioinformatic pipeline used, while we excluded the presence of the Burkholderia cepacia pathogen complex through OTU-, oligotyping-, and ASV-based analysis (68).

In summary, with the main purpose being to investigate the microbiological impact of this pregrinding decontamination treatment in the short and long term, we characterized the potentially active microbiota of two Piedmontese steak tartare production runs. Considering the different microbiota compositions of the two production runs from the early storage stages and their distinct final conditions of spoilage as proof-of-concept, we can reason that each production run might undergo faster or slower spoilage as a function of its initial metataxonomic profile. Therefore, more extensive investigations on a wide set of production runs at the RNA and DNA levels are now needed, in order to create a benchmark database of those profiles that can alternatively boost or slow down the spoilage dynamic of each product batch. This kind of approach will likely lead to the ability to predict and maximize the shelf life of highly perishable foods like ground beef.

## MATERIALS AND METHODS

### Meat processing, EW preparation, and experimental treatments.

The experiments were performed in a local processing plant (Piedmont, Italy) in which Piedmontese steak tartare is produced from three types of beef cuts (rump, thick flank, and sirloin) of adult females of the Piedmontese breed (Bos taurus; >30 months of age and >15 days of beef carcass aging). Briefly, the beef cuts are dissected from the carcass quarter, manually portioned into regular trimmings (∼500-g pieces), mixed, roughly ground by a single passage in a screw grinder (TCM grinder; Omet Foodtech srl, SI, Italy), and directly vacuum packed (120 ± 10 g each) in transparent linear low-density polyethylene bags (LLDPE) (oxygen transmission of 0.83 cm^3^·m^−2^·h^−1^ at 23°C, 30 cm by 30 cm) without operator handling or the addition of ingredients or preservatives. The expiration date after 14 days of vacuum storage at 4°C was fixed by the producer.

In this frame, experimental decontaminations of the meat trimmings with EW were performed *in situ* before the grinding step. The EW was freshly produced the day before the experiments in a nonmembrane generator (Eva system 100; De Nora S.P.A., Milan, Italy) by electrolysis of a 4-g/liter solution of KCl and diluted in distilled water to reach the desired final available free chlorine concentration (FCC), which was set to 100 ± 2.10 ppm (pH 8.55 ± 0.10, 11.5°C ± 1.0°C, oxidation reduction potential of 735 to 740) after preliminary *in vitro* evaluation of decontamination efficiency on meat trimmings. The analyses of FCC in EW and total chlorate residues in treated meat were performed by the Laemme Group (Tentamus Company, Moncalieri, Italy) following ISO 7393-2:2017 and ISO 10304-4:1997.

In practice, two production runs were followed, in July (production run A) and August (production run B) 2019, each constituted by two replicates (∼5 kg each; different carcass quarter of origin), equally divided into two parts, (i) a treated lot, in which meat trimmings were immerged in EW solution (wt/vol ratio of 1:10) for 90 s in a stainless-steel basket with continuous stirring, air dried for 1 min, and then ground, and (ii) an untreated control lot (Fig. S1). Surfaces in contact with meat trimmings (the inside of the meat grinder and the basket for the immersion) were cleaned and sanitized before the treatment and grinding of each lot by following the routine company procedures of an initial washing with high-pressure water, washing in foaming alkaline-free chlorine detergent (pH 13, >350 ppm free chlorine, 30 min), and a final rinse. To assess the cleaning-sanitizing efficacy and quantify the environmental contamination of meat contact surfaces, swabs were collected from the stainless-steel basket (*n* = 3) and from inside the meat grinder (*n* = 5) before the treatment of each lot. Knives, chopping boards, and the operator’s gloves were not considered for swabs since the cutting of meat trimmings was prior to the start of the experiment.

Meat trimmings were sampled by swabbing the surface before grinding (BG) (*n* = 3; 10 cm^2^) and immediately after grinding (ground beef [GB]) but before packaging. Around 40 vacuum packages were produced and stored at 4.0 ± 0.5°C without light exposure, and sampling was performed at 1, 7, 14, and 21 days. At each sampling point, two bags were sampled: one was immediately subjected to microbiological analysis, and one stored at −80°C for further physicochemical analysis.

### Microbiological analysis.

The swabs were supplemented with 5 ml of buffered peptone water (BPW), manually mixed for 2 min, and squeezed, extracting approximately 10 ml of suspension. Serial dilutions were set up from swab extracts and minced meat samples (10 g of meat in 90 ml of BPW), and microbial counts were performed as follows: total viable counts (TVC) of mesophilic bacteria on plate count agar incubated for 72 h at 30°C, *Brochothrix* spp. on STAA (streptomycin sulphate, thallous acetate, and actidione [cycloheximide]) medium incubated at 25°C for 48 h, Pseudomonas spp. on Pseudomonas agar base with cetrimide-fucidin-cephaloridine (CFC) selective supplement incubated at 25°C for 48 h, lactic acid bacteria (LAB) on De Man-Rogosa-Sharpe (MRS) agar incubated for 72 h at 30°C, and *Enterobacteriaceae* on violet red bile glucose agar (VRBGA) incubated for 24 h at 37°C. All media and supplements were provided by Biolife s.p.a. (Milan, Italy) unless otherwise stated.

An aliquot of 5 ml from each sample was centrifuged, and the pellet was collected and stored with RNAlater (Ambion; Thermo Scientific, Milan, Italy) at −80°C until RNA extraction and amplicon-based sequencing analysis were performed.

### Proximate composition, FAME pattern, and peroxide value.

The moisture content was determined using a Sartorius MA30 thermo-balance (Sartorius AG, Göttingen, Germany). The total nitrogen content and total protein content (conversion factor, 6.25) were obtained according to the Kjeldahl method, using Kjeltec system I (Foss Tecator AB, Höganäs, Sweden). The lipid fraction was determined on previously freeze-dried samples, using a semiautomatic Soxhlet Büchi extraction system B-811 (Büchi Labortechnik AG, Flawil, Switzerland) for 12 h, employing dichloromethane as the solvent.

Fatty acid methyl esters (FAMEs) were obtained by transesterification of triglycerides (200 μl) as previously described (69). FAMEs were analyzed on a Thermo Trace 1300 gas chromatograph (GC) equipped with a flame ionization detector (FID) and a split-splitless injector, using a DB23 column (30 m, inner diameter of 0.25 mm, and film thickness of 0.25 μm; J&W Scientific). Hydrogen was used as the gas carrier, with a flux of 1.5 ml/min. The injector and the detector were operated at 250°C and 350°C, respectively, and the temperature ramp was 5°C/min. The identification was obtained by comparing the retention times obtained from a mixture of 37 FAME standards (Supelco).

In order to determine the peroxide values of all the samples, a spectrophotometric method described previously (70) was applied. An amount of lipid of between 0.01 g and 0.3 g was weighed in a 10-ml glass tube, and 9.9 ml of chloroform-methanol mixture (7:3, vol/vol) and 50 μl of ammonium thiocyanate solution (30%, wt/vol) were added. The sample was vortexed for 5 s. Then, 50 μl of iron(II) chloride solution (2 mg/ml acidified with 2 μl of 10 M HCl) was added and the sample was vortexed for 5 s and incubated for 5 min at room temperature in the dark. Then, the absorbance was determined at 500 nm against a blank containing all reagents except the sample using a spectrophotometer (Shimadzu UV-1900).

### Analysis of VOCs. (i) SPME of VOCs.

Fiber coated with divinylbenzene/carboxen/polydimethylsiloxane (Supelco solid-phase microextraction [SPME] fiber assembly 50/30 μm DVB/CAR/PDMS) was used for the adsorption of volatile organic compounds. About 1.5 g of fresh sample was inserted into a 10-ml vial, and 50 μl of a 1-mg/liter camphor solution was added as the internal standard. The vial was then sealed with a cap fitted with a polytetrafluoroethylene (PTFE)/silicone pierceable septum. The prepared sample was incubated in a water bath at a temperature of 40°C for 15 min, after which the fiber was exposed in the headspace at a temperature of 40°C for 30 min.

### (ii) Detection of volatile organic compounds using GC-FID.

A Thermo TRACE 1300 gas chromatograph (GC) (Thermo Finningan, Rodano, Milan, Italy) equipped with a FID and a split-splitless injector was used. A low-polarity DB-5 Agilent Technologies column (30 m by 0.25 mm by 0.25 μm) (J&W Scientific, Folsom, CA) with a 95% dimethyl, 5% phenyl, polymethylsiloxane stationary phase was used. The injector and the detector were operated at 250°C and 350°C, respectively. Thermal desorption of the compounds from the SPME fiber was carried out in the splitless mode (split flow, 12.0 ml/min; splitless time, 2 min). Hydrogen was the carrier gas, with a constant flow rate of 1.2 ml/min. The oven was held at 50°C for 2 min and then heated to 220°C at a speed of 5°C/min and kept constant for 5 min. The overall timing of the analysis was 41 min. The VOC identification was obtained by comparison with the elution times and retention indexes of 11 standards (ethyl lactate, ethyl acetate, ethyl butyrate, ethyl hexanoate, benzaldehyde, hexanal, 1-pentanol, 1-hexanol, 2,3-butanedione, 3-methyl-1-butanol, and acetoin), chosen on the basis of the evidence of their significant changes in meat matrices subjected to various methods of conservation (25). The quantities of the volatiles were estimated by comparison of their peak areas with that of the camphor internal standard using area normalization.

### Amino acid and biogenic amine determination. (i) Preparations of samples and standards.

Eighty-five milliliters of ultrapure water (18.2 MΩ-cm at 25°C) was added to 10 g of lyophilized and defatted meat sample and the mixture homogenized for 2 min with an Ultra-Turrax (Ultra-Turrax T25 basic; IKA). Five milliliters of 100% trichloroacetic acid (TCA) (wt/vol) was added, and the mixture left to rest for 5 min. After filtration, an aliquot of 50 ml was extracted with ether (15 ml 3 times) in order to remove lipids and excess TCA. The aqueous solution (after removing traces of ether in a Rotovapor) was adjusted to 50 ml with ultrapure water and filtered through a 0.45-μm MS mixed cellulose ester (MCE) syringe filter before high-performance liquid chromatography (HPLC) analysis. The standard solutions of amines and precursor amino acids were prepared by dissolving each compound in HPLC-grade water.

### (ii) Chromatographic conditions.

For the chromatographic analyses, a validated ion-pair HPLC method was applied (71), with slight modifications. A C_18_ reverse-phase Spherisorb S5 ODS 2 column (Phase Separation, Inc., Deeside, Clwyd, United Kingdom) (250-mm by 4.6-mm inner diameter, particle size 5 μm) was used. The ion pair reagent, heptanesulfonate/ortho-phosphate, was prepared by dissolving heptanesulfonate and phosphate (KH_2_PO_4_) in ultrapure water and adjusting the pH to 3.5 with *ortho*-phosphoric acid; octylamine (another ion-pairing reagent) was used at a low concentration (20 μl/liter solution) as a second ion-pairing reagent (eluant 1). Methanol was employed as an organic modifier (eluant 2). Mobile phases were filtered and degassed before use. Conditions were as follows. Pump A, eluant 1 (heptanesulfonate, 10 mM; phosphate, 10 mM). Pump B, HPLC-grade methanol. Gradients: 100% pump A for 1 min; pump B from 0% to 26% in 5.25 min; pump B from 26% to 35% in 9 min; pump B from 35% to 42% in 1.5 min; pump B at 42% for 24 min; pump A at 100% for 10.40 min. Rate of flow, 1 ml/min. Detection (UV and diode-array detection [DAD]) at 215 nm. The column was kept at 27°C during the analyses (71).

### RNA extraction, cDNA synthesis, and amplicon-based sequencing.

Total RNA was extracted using the MasterPure complete DNA and RNA purification kit (Epicentre, Madison, WI, USA) according to the manufacturer’s instructions. Three microliters of Turbo *DNase* (Ambion) were added to digest the DNA in the RNA samples, with an incubation of 3 h at 37°C. The complete denaturation of genomic DNA (gDNA) was confirmed by PCR amplification of the partial 16S rRNA gene, using forward primer FD1 and reverse primer RD1 (72). The quality of the extracted RNA was evaluated and quantified using a NanoDrop spectrophotometer (Thermo Scientific, Milan, Italy). Two samples of production run B were excluded due to the low RNA quality and amount (from ground beef at 0 and 14 days). The cDNA was synthesized from 2 μg of RNA with the Moloney murine leukemia virus (M-MLV) reverse transcriptase system (Promega, Milan, Italy), and a library of the V3-V4 region was constructed from the 16S rRNA gene region using previously described primers and conditions (25).

The PCR products were purified by means of an Agencourt AMPure kit (Beckman Coulter, Milan, Italy), and the resulting products were tagged with sequencing adapters using the Nextera XT library preparation kit (Illumina, Inc., San Diego, CA) according to the manufacturer’s instructions. Sequencing was performed using a MiSeq Illumina instrument (Illumina) with V3 chemistry, which generated 2 × 250-bp paired-end reads. MiSeq Control Software version 2.3.0.3, RTA version 1.18.42.0, and CASAVA version 1.8.2 were used for the base-calling and Illumina barcode demultiplexing processes.

### Bioinformatic analysis.

A total of 4,397,672 raw reads obtained from 46 samples were subsequently processed through two bioinformatic pipelines to obtain, in parallel, amplicon sequence variants (ASVs) with the divisive amplicon denoising algorithm (DADA2) and operational taxonomic units (OTUs) through *de novo* clustering at 97%.

To obtain the ASVs, the reads in FASTQ format were analyzed through the DADA2 package in the R environment (73). Briefly, 2,821,250 single-end reads passed the quality-filtering parameters applied [truncLen=c(245,240); trimLeft = c(20); maxN = 0; maxEE=c(2); truncQ = 5; minLen = c(50)] with an average value of 61,332 reads/sample and thus were merged (minimum overlap of 20 bp) and subjected to *de novo* chimera removal (per-sample method; 9.7% of merged sequences were detected as chimeras and removed). OTU clustering was performed from paired-end reads assembled with FLASH software (74) and further quality filtered (at Phred < Q20) with QIIME 1.9.0 soſtware (75). Chimeras were removed through VSEARCH software (https://github.com/torognes/vsearch), and OTUs were picked at a 97% similarity threshold by UCLUST algorithms (76). A total of 3,529,815 reads passed the filters applied by QIIME (68,526 reads/sample).

ASV taxonomy was assigned at 99% sequence similarity through the Bayesian classifier method (77) by using the 2019 release Silva reference database of 16S rRNA for bacterial ASVs (https://www.arb-silva.de/documentation/release-138). To assign the OTU taxonomy, the centroid sequences of each cluster were matched at 97% similarity to the 2013 version of the Greengenes 16S rRNA gene database (GG97; https://drive5.com/usearch/manual/download_gg97.html), as suggested for this approach. ASVs were also aligned to the GG97 database as a cross-check, while the alignment of representative OTUs against the 99% Silva database was not performed due to the lower percentage of OTU clustering used (97%). Both ASV and representative OTU sequences were aligned with PyNAST (78), and unrooted phylogenetic trees were constructed with FastTree (79) to allow further phylogenetic analysis.

Within-OTU diversity was resolved by using an oligotyping procedure described previously (80). Briefly, all reads assigned to the most abundant OTUs were extracted and shortened to 445 bp by two trimming steps, as follows: (i) the length was shortened to 457 bases and (ii) the first 13 bases were removed from the 3′ end. Shannon’s entropy analysis was performed in order to identify positional variations at the single-nucleotide level. The list of OTUs oligotyped (taxonomic assignment), initial number of reads, average Shannon’s entropy values, high entropy positions chosen (-C option; Shannon’s entropy >0.2), and minimum substantive abundances (-M option) are summarized in Table S5. Moreover, each oligotype was required to appear in at least one sample at 1.0% of abundance (-a option) to reduce the noise generated by low-abundance oligotypes. BLASTn (https://blast.ncbi.nlm.nih.gov/Blast.cgi) was used to query the representative oligotype sequences against the NCBI database, and the top hits (higher percent identities) were considered for taxonomic assignment at the species level; when multiple assignments at the species level were obtained, the oligotype taxonomy was assigned at the genus level.

Metagenome inference was performed with PICRUSt (81): gene family abundances were predicted, and KEGG orthologs were then collapsed at level 3 of the hierarchy.

### Statistics.

Statistical analyses and data plotting were performed using R program for Statistical Computing 4.1.0 (http://www.r-project.org).

Normality and homogeneity of the data (Log-transformed abundances, viable counts, and BA, FAA, and VOC concentrations) were checked by means of Shapiro-Wilk’s W and Levene’s tests, respectively. To assess the overall variation and differences between multiple groups, one-way analysis of variance (ANOVA) coupled with Tukey’s *post hoc* test was carried out for parametric data. For nonparametric data, the Kruskal-Wallis test was used to assess the overall variation and differences between multiple groups, whereas Wilcoxon’s test was used to compare individual groups. For the PCA, the data were scaled to unit variance and the *FactoMineR* package was employed.

Alpha diversity metrics (observed species, abundance-based coverage estimator [ACE], Shannon, Simpson, Fisher, and phylogenetic diversity [PD] whole tree) and weighted UniFrac beta-diversity distance were calculated with the *phyloseq* package (82) from rarefied OTU and ASV tables (the rarefaction limit was determined by the sample containing the lowest number of sequences) and the respective phylogenetic trees when needed. Permutational analysis of variance (PERMANOVA) was conducted on the original ASV and OTU frequency tables to quantitatively evaluate the effects of time, production run, and treatment (individually or interactively) on the variation of bacterial community composition (relative abundances) by using the ADONIS function based on 999 permutations and Bray-Curtis dissimilarity distance.

To construct the oligotype cooccurrence network, the SparCC algorithm (83) was run with default parameters and 100 bootstraps using the R package *SpiecEasi*. Pseudo-*P* values were calculated as the proportion of simulated bootstrapped datasets; only highly significant positive correlations were used to infer the network (SparCC correlation of >0.6 and *P* values of <0.001). The oligotype network was visualized using the program Gephi 0.9.2-beta (https://gephi.org), and the presence of recurrent subnetwork modules (groups of oligotypes that are covarying) was detected through the algorithm described in reference 84.

Metataxonomic data (OTU, ASV, and oligotype abundances) and viable counts were correlated with chemical data (AB, FAA, and VOCs) by means of Pearson’s product-moment correlation and Spearman’s rank correlation, respectively. An abundance table of predicted metagenomes was imported in the *GAGE* Bioconductor package (85) to identify biological pathways significantly (*P < *0.001) overrepresented or underrepresented in the two treatment lots (control and EW treated) and production runs (A and B).

### Data availability.

Sequencing data were deposited at the Sequence Read Archive of the National Center for Biotechnology Information under BioProject accession number PRJNA734136.
