# Supplementary material for: Impact of Electrolyzed Water on the Microbial Spoilage Profile of Piedmontese Steak Tartare
Source: Microbiol Spectr. 2021 Nov 17;9(3):e01751-21. doi: 10.1128/Spectrum.01751-21 (PMC8597643; doi:10.1128/Spectrum.01751-21)
Supplement: SUPPLEMENTAL FILE 1 — Supplemental material. Download SPECTRUM01751-21_Supp_1_seq9.pdf, PDF file, 2 MB [file spectrum01751-21_supp_1_seq9.pdf]

Impact of electrolyzed water on the microbial spoilage profile of Piedmontese steak tartare

Botta C., Coisson J. D., Ferrocino I., Colasanto A., Pessione A., Cocolin L., Arlorio M., Rantsiou K.

Supplemental material for publication

Supplementary Figure 1. Total Viable Counts (TVC) enumerated on meat trimming surfaces before and after 90 seconds of immersion in EW at 25, 50 and 100 ppm of FCC. Significant differences are displayed (Student's T-test).

Supplementary Figure.2. Schematic layout of the experimental plan: in each production two replicates (meat trimmings from two different quarters) were treated and followed along the shelf-life, together with the untreated control. Samplings were performed the day of production before grinding (BG) and ground beef (GB); during the 21 days of vacuum storage at 4 °C.

Supplementary Figure 3. Treated and untreated (control) beef trimmings and ground beef.

Supplementary Figure 4. Bidimensional representation of the Principal Component Analysis on the meat samples centesimal composition and FAMEs pattern data (on the left score plot and on the right loadings plot) (C: control, EW: treated; the numbers are the days of storage); PCA model was obtained with two first principal components that explained 72,84 % of total response variance.

Supplementary Figure 5. Oligotypes co-occurrence network based on SparCC correlation algorithm (100 bootstraps); only signific positive correlation between oligotypes absolute frequencies are displayed (SparCC correlation > 0.6 and *P*-values < 0.001). Nodes are made proportional to the oligotype occurrence and coloured in relation to the species of belonging (caption). Edges thicknesses are made proportional to SparCC correlation value (from 0.6 to 1); lengths have no specific meaning. Modules of nodes (clusters of co-occurring oligotypes) are highlighted with dashed circles and coded (refer to Table 1 for codes and modules composition).

Supplementary Figure 6. Stacked bar plots comparing microbiota composition (relative abundance in %) displayed at the species level (colour coding key) obtained from ASVs- based approaches and oligotyping of the most abundant OTUs (genus level); samples are sequentially grouped according to the sampling points (Before Grinding [BG], Ground Beef [GB] and storage days [T]), thesis (control, EW) and productions (A, B).

S_Figure 1

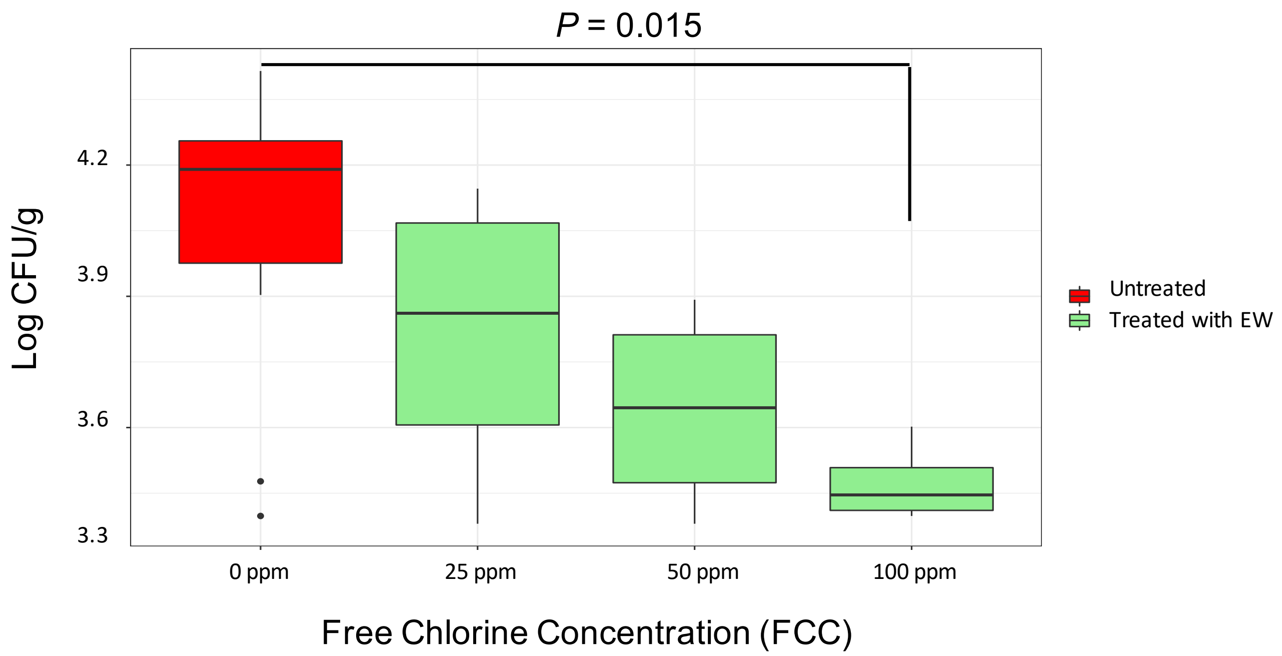

S_Figure 2

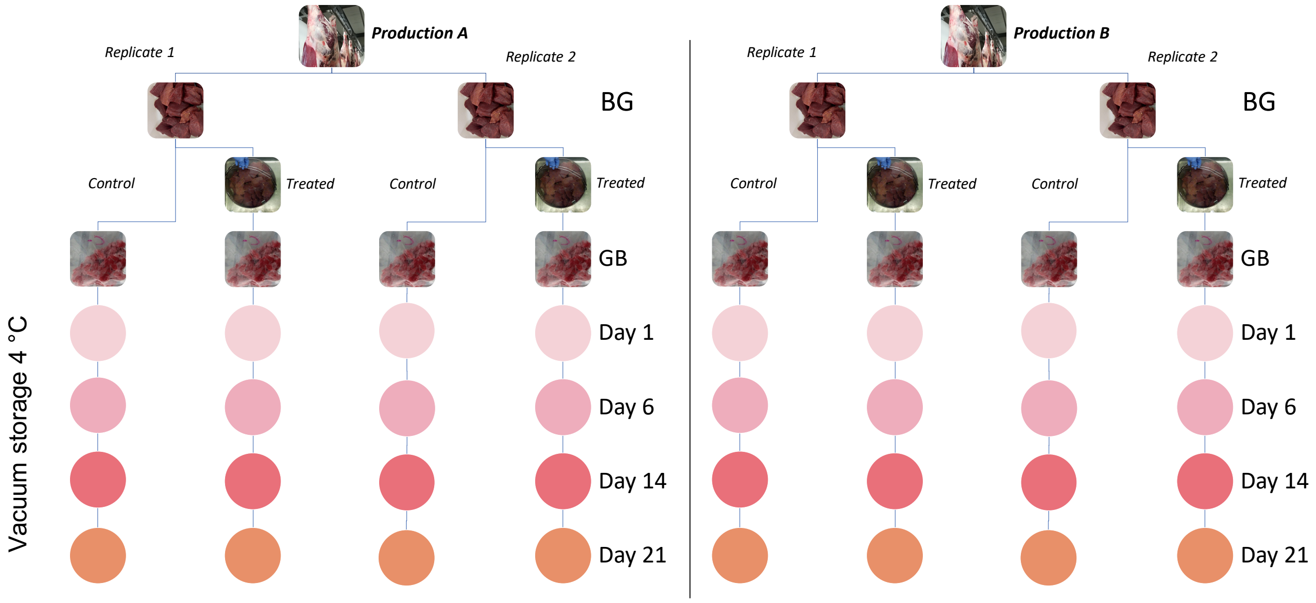

S_Figure 3

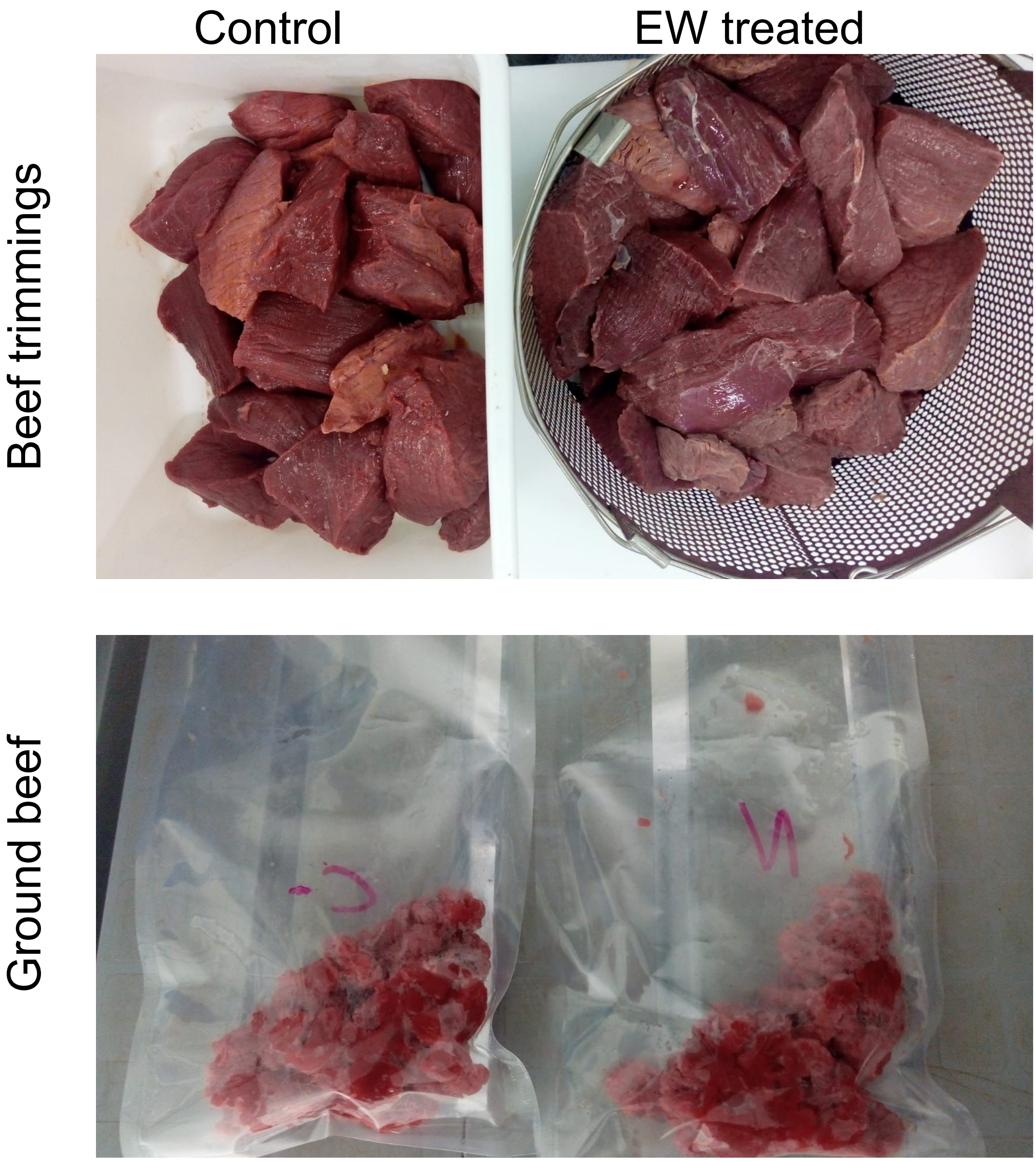

S_Figure 4

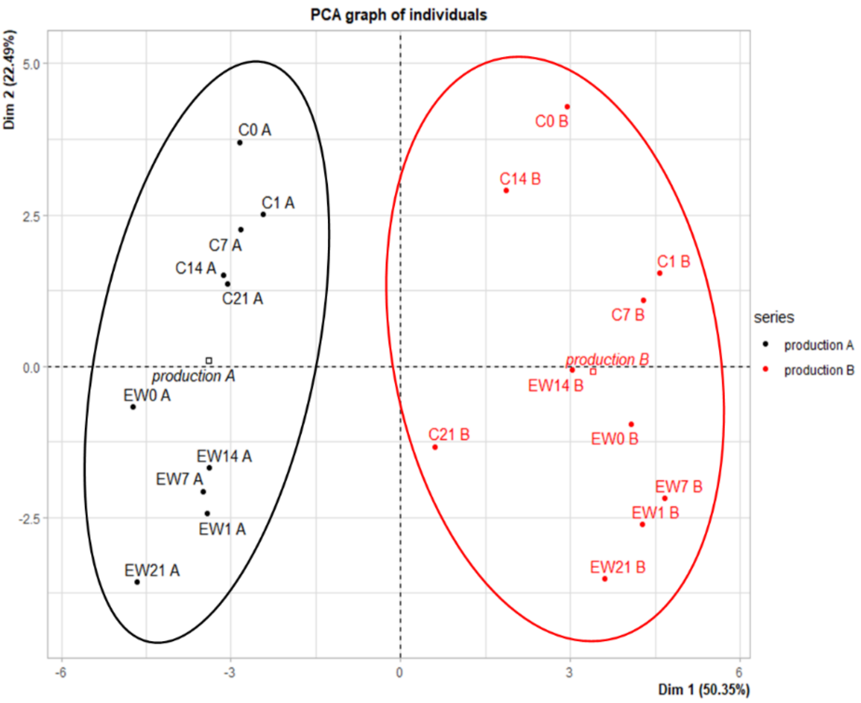
PCA graph of individuals
Dim 2 (22.49%)
Dim 1 (50.35%)
5.0
2.5
0.0
-2.5
-6
-3
0
3
6
C0 A
C1 A
C7 A
C14 A
C21 A
production A
EW0 A
EW14 A
EW7 A
EW1 A
EW21 A
C0 B
C14 B
C1 B
C7 B
production B
EW14 B
C21 B
EW0 B
EW7 B
EW1 B
EW21 B
series
production A
production B

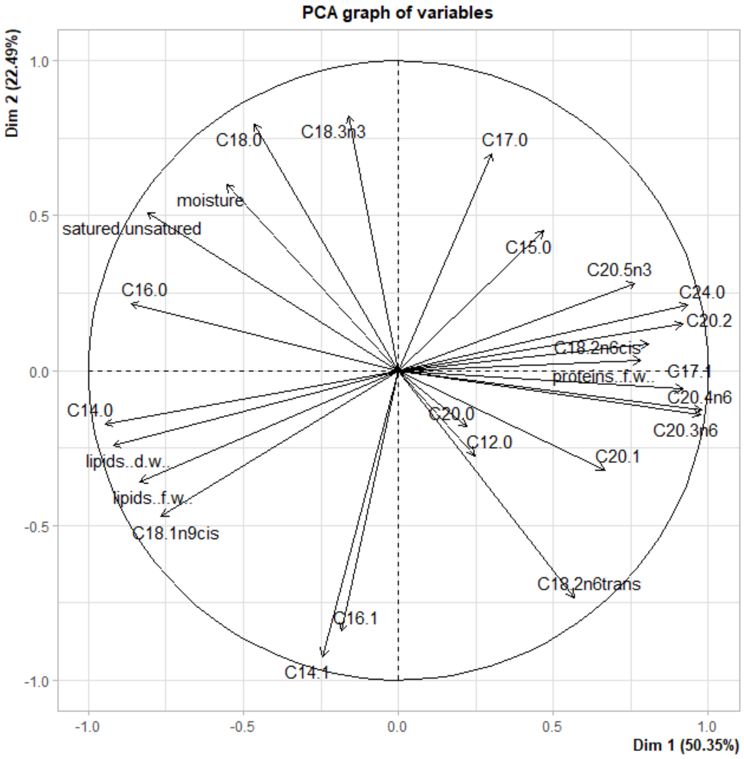
PCA graph of variables
Dim 2 (22.49%)
Dim 1 (50.35%)
1.0
0.5
0.0
-0.5
-1.0
-1.0
-0.5
0.0
0.5
1.0
C18.0
C18.3n3
C17.0
moisture
satured/unsatured
C15.0
C20.5n3
C16.0
C24.0
C20.2
C18.2n6cis
C17.1
proteins..f.w..
C20.4n6
C14.0
C20.0
C20.3n6
C12.0
lipids..d.w..
C20.1
lipids..f.w..
C18.1n9cis
C18.2n6trans
C16.1
C14.1

S_Figure 5

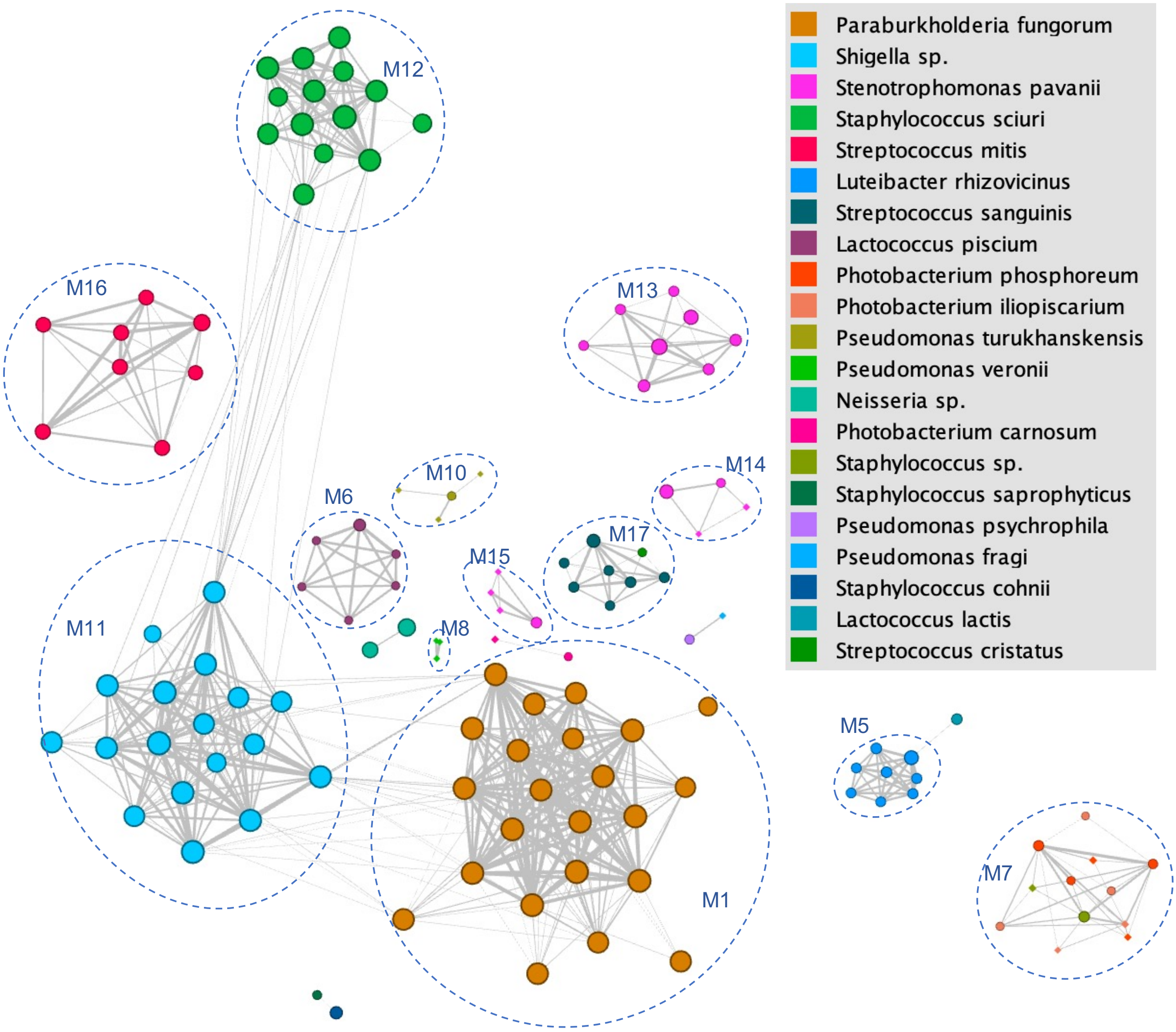

M12
M16
M13
M10
M14
M6
M17
M15
M11
M8
M1
M5
M7
Paraburkholderia fungorum
Shigella sp.
Stenotrophomonas pavanii
Staphylococcus sciuri
Streptococcus mitis
Luteibacter rhizovicinus
Streptococcus sanguinis
Lactococcus piscium
Photobacterium phosphoreum
Photobacterium iliopiscarium
Pseudomonas turukhanskensis
Pseudomonas veronii
Neisseria sp.
Photobacterium carnosum
Staphylococcus sp.
Staphylococcus saprophyticus
Pseudomonas psychrophila
Pseudomonas fragi
Staphylococcus cohnii
Lactococcus lactis
Streptococcus cristatus

S_Figure 6

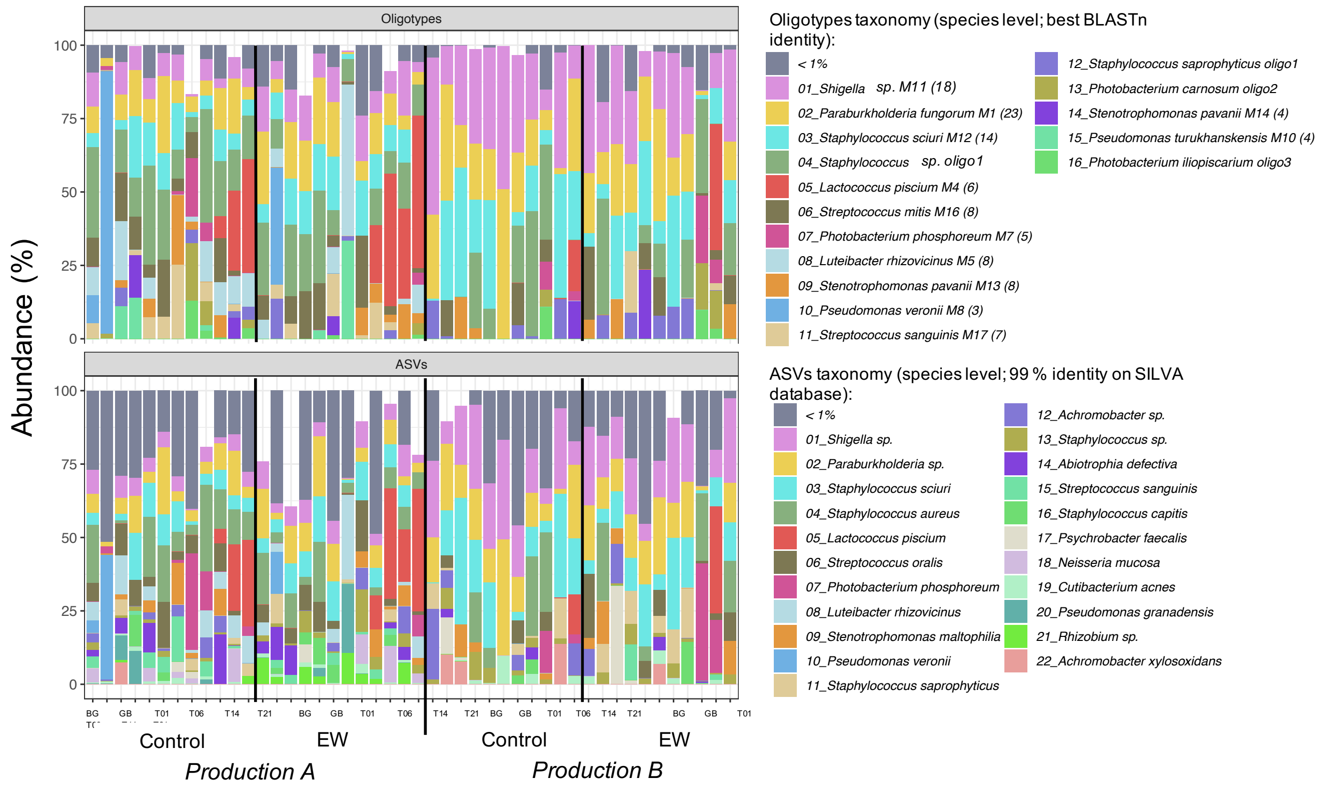
Oligotypes
ASVs
Abundance (%)
100
75
50
25
0
BG
GB
T01
T06
T14
T21
Control
EW
Production A
Production B
Oligotypes taxonomy (species level; best BLASTn identity):
< 1%
01_Shigella sp. M11 (18)
02_Paraburkholderia fungorum M1 (23)
03_Staphylococcus sciuri M12 (14)
04_Staphylococcus sp. oligo1
05_Lactococcus piscium M4 (6)
06_Streptococcus mitis M16 (8)
07_Photobacterium phosphoreum M7 (5)
08_Luteibacter rhizovicinus M5 (8)
09_Stenotrophomonas pavanii M13 (8)
10_Pseudomonas veronii M8 (3)
11_Streptococcus sanguinis M17 (7)
12_Staphylococcus saprophyticus oligo1
13_Photobacterium carnosum oligo2
14_Stenotrophomonas pavanii M14 (4)
15_Pseudomonas turukhanskensis M10 (4)
16_Photobacterium iliopiscarium oligo3
ASVs taxonomy (species level; 99 % identity on SILVA database):
< 1%
01_Shigella sp.
02_Paraburkholderia sp.
03_Staphylococcus sciuri
04_Staphylococcus aureus
05_Lactococcus piscium
06_Streptococcus oralis
07_Photobacterium phosphoreum
08_Luteibacter rhizovicinus
09_Stenotrophomonas maltophilia
10_Pseudomonas veronii
11_Staphylococcus saprophyticus
12_Achromobacter sp.
13_Staphylococcus sp.
14_Abiotrophia defectiva
15_Streptococcus sanguinis
16_Staphylococcus capitis
17_Psychrobacter faecalis
18_Neisseria mucosa
19_Cutibacterium acnes
20_Pseudomonas granadensis
21_Rhizobium sp.
22_Achromobacter xylosoxidans

S Table 1. Concentrations (µg/kg) of the eleven Volatile Organic Compounds (VOCs) detected in the headspace of steak tartare during shelf-life. Data (mean ± SD) are separately shown for each thesis (Control, EW) in each production (A, B); different lowercase letters (a, b, c) and different uppercase letters (A, B, C) indicate significance differences between sampling points (day 0, 7, 21) and thesis, respectively (*P* <0.05; ANOVA and Tukey's post-hoc test).

S Table 2. Concentrations (mg/kg) of Free Amino Acids (FAA) and Biogenic Amines (BA) detected in the steak tartare during shelf-life. Data (mean ± SD) are separately shown for each thesis (Control, EW) in each production (A, B); different lowercase letters (a, b, c) and different uppercase letters (A, B, C) indicate significance differences between sampling points (day 0, 1, 7, 14, 21) and thesis, respectively (*P* <0.05; ANOVA and Tukey's post-hoc test).

S Table 3. Concentrations (meq O2/Kg) of peroxides detected in the steak tartare during shelf-life. Data (mean ± SD) are separately shown for each thesis (Control, EW) in each production (A, B); different lowercase letters (a, b, c) and different uppercase letters (A, B, C) indicate significance differences between sampling points (day 0, 1, 7, 14, 21) and thesis, respectively (*P* <0.05; ANOVA and Tukey's post-hoc test).

S Table 4. Comparison of ASVs- and OTUs- based approaches at the higher taxonomic resolution reachable. Asterisks (*) indicate for ASVs a taxonomic assignment at 100 % of similarity to Silva DB.

S Table 5. Table displaying the OTUs subjected to oligotyping and parameters used for the analysis.

S Table 1

		Production A						Production B					
		Day 0		Day 7		Day 21		Day 0		Day 7		Day 21	
		Control	EW treated	Control	EW treated	Control	EW treated	Control	EW treated	Control	EW treated	Control	EW treated
Alcohols	1-Hexanol	29.4bA ± 3.33	12.7bB ± 2.05	34.2bA ± 12.0	36.5bA ± 8.48	471^{aB} ± 160	1341^{aA} ± 218	20.6aB ± 9.38	69.0aA ± 8.38	25.9aA ± 16.8	15.0aA ± 5.93	58.6aA ± 2.40	472^{aA} ± 206
	1-Pentanol	16.7bA ± 0.26	15.26bA ± 2.01	60.0aA ± 2.91	24.4bB ± 6.13	43.8abA ± 13.9	57.1aA ± 6.48	28.6aA ± 14.3	86.5aA ± 53.0	10.7aA ± 0.76	14.0aA ± 2.46	15.8aA ± 4.69	20.3aA ± 7.03
	Isoamyl alcohol	97.0aA ± 5.49	45.8aA ± 16.1	27.2bB ± 2.83	77.0aA ± 7.19	35.0bA ± 1.32	91.7aA ± 32.8	41.4aB ± 23.9	242aA ± 16.5	39.6aA ± 8.63	23.5bA ± 4.72	12.0aB ± 1.84	32.3bA ± 6.35
Ketones	Diacetyl	0.28bA ± 0.01	0.48bA ± 0.20	2.53bA ± 0.38	3.12bA ± 0.99	53.5aA ± 2.63	74.2aA ± 21.5	2.10aA ± 0.59	8.28bA ± 5.42	1.59aB ± 0.23	42.2abA ± 1.87	12.2aB ± 11.6	79.3aA ± 23.7
	Acetoin	7.03aA ± 2.24	8.09bA ± 0.60	3.82aB ± 0.99	12.8bA ± 1.95	4.09aA ± 0.15	27.6aB ± 6.99	9.50aA ± 3.21	20.9aA ± 4.40	6.87aA ± 3.05	11.9bA ± 1.08	7.19aA ± 3.79	5.78bA ± 1.72
Aldehydes	Benzaldehyde	0.00bA ± 0.00	0.00bA ± 0.00	0.00bB ± 0.00	13.9bA ± 1.54	70.0aA ± 14.5	192aA ± 85.6	0.00aA ± 0.00	0.00bA ± 0.00	0.00aA ± 0.00	0.00bA ± 0.00	15.6aB ± 10.9	72.6aA ± 2.03
	Hexanal	459aA ± 78.5	525bA ± 94.2	806^{aA} ± 261	504bA ± 58.4	1436^{aA} ± 860	2751^{aA} ± 561	48.9aA ± 24.8	284aA ± 80.8	469^{aA} ± 241	469^{aA} ± 124	175aA ± 89.1	235aA ± 22.9
Ethers	Ethyl acetate	1.66aA ± 0.08	1.41bA ± 0.35	0.90aA ± 0.06	2.61abA ± 0.90	49.1aA ± 27.0	15.0aA ± 7.31	0.96aA ± 0.16	3.34aA ± 2.27	2.16aA ± 0.91	21.1aA ± 7.69	3.28aA ± 2.20	46.7aA ± 23.8
	Ethyl hexanoate	71.2bA ± 65.7	36.3bA ± 9.49	93.2bA ± 53.6	58.4bA ± 10.6	263aA ± 1.63	292aA ± 13.4	110bB ± 66.5	334aA ± 17.7	1851^{aA} ± 847	1017^{aA} ± 435	2582^{aA} ± 235	1582^{aA} ± 660
	Ethyl butyrate	23.9aA ± 10.4	24.0aA ± 9.03	406^{aA} ± 377	162aA ± 91.1	250aA ± 73.9	150aA ± 6.06	17.8aA ± 4.71	42.2aA ± 25.9	59.8aA ± 20.1	51.2aA ± 24.5	41.9aA ± 9.91	31.8aA ± 5.19
	Ethyl lactate	2.92aA ± 0.28	0.97aA ± 0.86	5.95aA ± 2.30	1.98aA ± 0.87	5.18aA ± 0.72	3.33aA ± 2.82	2.20bA ± 0.26	5.36aA ± 5.29	2.17bB ± 0.07	17.9aA ± 1.84	19.9aA ± 4.17	11.0aA ± 5.52

S Table 2

	Time	TYROSINE		HISTIDINE		PHENYLALANINE		TRIPTOPHAN		TYRAMINE		HISTAMINE		2-PHENYLETHYAMINE		TRIPTAMINE	
		Control	EW treated	Control	EW treated	Control	EW treated	Control	EW treated	Control	EW treated	Control	EW treated	Control	EW treated	Control	EW treated
Production A	Day 0	71.3ab ± 12.7	46.9b ± 5.90	31.3c ± 5.71	19.0d ± 2.86	291c ± 53.9	192c ± 26.3	6.11b ± 1.31	5.68b ± 0.75	n.d.	n.d.	7.06a ± 0.78	4.90a ± 1.30	n.d.	n.d.	0.47c ± 0.28	0.14c ± 0.04
	Day 1	61.8b ± 2.93	48.3b ± 4.19	28.9c ± 1.36	21.7d ± 1.84	331bc ± 4.98	307b ± 16.7	7.31b ± 0.59	6.12b ± 0.57	n.d.	n.d.	5.28b ± 0.53	3.86ab ± 0.25	n.d.	n.d.	0.11c ± 0.01	0.12c ± 0.03
	Day 7	82.2a ± 2.76	70.7a ± 2.74	32.0c ± 1.36	29.1c ± 1.19	352ab ± 11.2	309b ± 33.2	12.2a ± 0.60	9.60a ± 1.30	5.75c ± 0.55	6.25c ± 0.91	3.20c ± 0.36	3.54b ± 0.33	n.d.	n.d.	0.32c ± 0.11	0.34c ± 0.03
	Day 14	14.5c ± 0.58	10.3c ± 0.11	41.1b ± 1.90	37.2b ± 0.70	287c ± 8.05	291b ± 4.37	1.45c ± 0.10	0.63c ± 0.06	94.5b ± 2.31	87.4b ± 0.48	2.36c ± 0.16	3.16b ± 0.40	n.d.	n.d.	7.64b ± 0.78	8.32b ± 0.43
	Day 21	7.61c ± 0.15	5.17c ± 0.07	50.3a ± 3.54	47.1a ± 0.74	400a ± 20.3	390a ± 9.43	0.38c ± 0.12	0.54c ± 0.08	137a ± 3.64	127a ± 1.12	0.85d ± 0.25	0.54c ± 0.28	n.d.	n.d.	12.1a ± 0.68	10.5a ± 0.20
Production B	Day 0	70.7a ± 15.2	57.5a ± 6.87	29.7c ± 6.18	24.9c ± 3.70	244a ± 33.8	225a ± 25.3	8.53a ± 1.82	7.80a ± 1.23	n.d.	n.d.	16.0a ± 2.11	14.3a ± 1.80	n.d.	n.d.	1.07d ± 0.53	1.34d ± 0.12
	Day 1	62.5a ± 5.04	56.1a ± 3.56	26.8c ± 2.26	23.9c ± 0.65	227a ± 19.2	216a ± 7.48	8.52a ± 0.86	7.05a ± 0.26	n.d.	n.d.	15.8a ± 0.87	13.3a ± 3.22	n.d.	n.d.	1.30d ± 0.10	1.36d ± 0.17
	Day 7	47.7b ± 6.04	18.0b ± 1.29	44.4b ± 4.50	47.7b ± 1.98	99.4c ± 5.97	104d ± 5.87	5.48a ± 1.30	1.97b ± 0.33	60.5c ± 7.43	88.9b ± 4.71	8.05c ± 0.42	5.88b ± 0.61	n.d.	n.d.	5.29c ± 0.46	8.66c ± 0.53
	Day 14	7.17c ± 0.63	4.63c ± 0.22	61.6a ± 2.47	54.6a ± 1.64	166b ± 3.88	146c ± 8.89	0.67c ± 0.03	0.43c ± 0.03	142b ± 3.09	139a ± 4.89	11.7b ± 0.45	11.9a ± 0.42	n.d.	n.d.	13.2b ± 0.28	12.4b ± 0.38
	Day 21	2.76c ± 0.28	1.84c ± 0.33	55.2a ± 3.18	48.3a ± 3.18	187b ± 4.16	169b ± 11.3	0.27c ± 0.09	0.27c ± 0.12	157a ± 9.18	147a ± 8.39	13.5b ± 1.22	14.4a ± 1.12	n.d.	n.d.	16.0a ± 2.02	13.8a ± 0.33

S Table 3

Peroxide value (*meq* O_2/*Kg*)				
	Production A		Production B	
	Control	EW treated	Control	EW treated
**Day 0**	2.72a ± 0.63	6.76a ± 1.38	17.7b ± 1.42	17.8b ± 2.16
**Day 1**	2.83a ± 0.55	3.88b ± 0.47	20.3a ± 2.54	23.9a ± 1.71
**Day 7**	1.57c ± 0.37	3.95b ± 0.86	2.47c ± 0.39	1.63c ± 0.54
**Day 14**	1.67bc ± 0.28	1.45c ± 0.23	3.18c ± 0.48	2.77c ± 0.75
**Day 21**	2.48ab ± 0.28	3.77b ± 0.55	3.22c ± 0.89	3.76c ± 0.86

S Table 4

GreenGenes (https://drive5.com/usearch/manual/download_gg97.htm)				Silva DB (https://www.arb-silva.de/documentation/release-138)	
OTUs (97 % clustering)		ASVs		ASVs	
**Taxa**	**Average abundance**	**Taxa**	**Average abundance**	**Taxa**	**Average abundance**
Shigella sp.	12.4	*Shigella sp.*	11.4	*Shigella sp.*	12.15
Staphylococcus sciuri	10.6	*Burkholderia sp.*	11.1	*Paraburkholderia sp.*	11.64
Bacilli [Class]	10.5	*Staphylococcus sciuri*	10.3	*Staphylococcus sciuri*	10.25
Burkholderia bryophila	9.5	*Staphylococcus aureus*	7.9	*Staphylococcus aureus*	7.90
Streptococcus sp.	6.6	*Lactococcus sp.*	4.7	*Lactococcus piscium**	4.71
Lactococcus sp.	5.3	*Streptococcus infantis*	4.1	*Streptococcus oralis*	4.14
Photobacterium angustum	4.5	*Staphylococcus sp.*	2.5	*Staphylococcus sp.*	2.96
Stenotrophomonas geniculata	4.3	*Achromobacter sp.*	2.4	*Stenotrophomonas maltophilia*	2.75
Bacillales [Order]	2.9	*Luteibacter rhizovicinus*	2.3	*Achromobacter sp.*	2.58
Achromobacter sp.	2.6	*Stenotrophomonas sp.*	1.8	*Staphylococcus saprophyticus*	2.47
Luteibacter rhizovicinus	2.4	*Abiotrophia sp.*	1.6	*Luteibacter rhizovicinus**	2.33
Burkholderia sp.	1.8	*Photobacterium angustum*	1.5	*Pseudomonas veronii*	2.22
Pseudomonas sp.	1.8	*Streptococcus sp.*	1.5	*Rhizobium sp.*	1.88
Pseudomonas veronii	1.5	*Photobacterium angustum*	1.4	*Abiotrophia defectiva**	1.71
Propionibacterium acnes	1.5	*Staphylococcus sp.*	1.3	*Photobacterium phosphoreum**	1.55
Enhydrobacter sp.	1.2	*Psychrobacter pulmonis*	1.3	*Streptococcus sanguinis*	1.47
Corynebacterium sp.	1.2	*Neisseria sp.*	1.1	*Photobacterium phosphoreum*	1.40
Lactobacillus sp.	1.2	*Propionibacterium acnes*	1.1	*Staphylococcus capitis*	1.32
Abiotrophia sp.	1.2	*Pseudomonas sp.*	1.0	*Psychrobacter faecalis*	1.31
Staphylococcus	1.2	*Achromobacter sp.*	1.0	*Neisseria mucosa*	1.09
Streptophyta sp.	1.2			*Cutibacterium acnes*	1.07
Neisseria sp	1.0			*Achromobacter xylosoxidans*	1.05
				Pseudomonas granadensis	1.04

S Table 5

Reads analysed	Shannon's entropy (no. of position > 0.2)	High entropy positions chosen (-C option)	M option	% of reads represented	Purity score
359,026	8	59, 102, 117, 123, 245, 247, 252, 267, 329, 348, 364, 381	50	96.61	0.34
82,183	12	64, 65, 71, 87, 101, 104, 108, 117, 118, 121, 122, 139, 188, 238, 246, 293, 294, 296, 315, 328, 380, 383, 389, 424	50	78.70	0.86
317,031	102	184, 189, 239, 244, 246, 248, 256, 286, 336, 379, 391, 394, 395, 396, 397, 398, 399, 401, 403, 404, 405, 406, 407, 408, 409, 410, 416, 418, 423, 424, 425, 429, 430, 433, 439, 440	50	91.44	0.61
135,369	25	88, 104, 105, 106, 117, 118, 120, 121, 122, 129, 135, 139, 150, 240, 245, 249, 252, 260, 277, 292, 308, 328, 389, 422,	50	89.51	0.72
168,450	83	92, 100, 106, 119, 124, 166, 201, 225, 236, 237, 244, 248, 249, 266, 271, 272, 278, 281, 293, 296, 304, 318, 326, 327, 332, 333, 338, 339, 343, 344, 346, 347, 348, 351, 352, 353, 354, 355, 356, 363, 364, 369, 372, 373, 378, 386, 389, 390, 394, 395, 396, 397, 398, 399, 400, 401, 402, 403, 404, 405, 406, 407, 408, 409, 410, 411, 415, 416, 417, 418, 422, 423, 424, 425, 428, 429, 430, 433, 436, 437, 438, 439, 440	10	78.51	0.73
103,259	96	25, 30, 68, 100, 102, 103, 104, 106, 107, 108, 109, 115, 116, 117, 118, 119, 120, 121, 122, 123, 159, 160, 183, 184, 237, 245, 246, 247, 248, 249, 256, 260, 265, 266, 277, 281, 283, 284, 292, 293, 294, 295, 296, 308, 316, 327, 364, 366, 367, 368, 369, 372, 373, 375, 378, 379, 380, 382, 383, 386, 389, 390, 391, 392, 393, 394, 395, 396, 397, 398, 399, 401, 402, 403, 405, 408, 409, 410, 411, 414, 416, 417, 418, 422, 423, 424, 425, 429, 430, 432, 433, 435, 437, 438, 439, 440	50	70.56	0.93
389,968	15	246, 256, 284, 389, 395, 397, 399, 404, 408, 409, 424, 430, 433, 439, 440	50	97.20	0.43
390,629	22	64, 65, 71, 87, 101, 104, 108, 117, 118, 121, 122, 139, 188, 238, 244, 246, 293, 315, 328, 380, 383, 424	50	89.88	0.44
151,145	9	106, 117, 238, 244, 293, 294, 322, 401, 42	50	70.95	0.95
179,580	20	8, 12, 37, 38, 43, 44, 114, 148, 206, 213, 251, 256, 258, 266, 275, 280, 290, 291, 307, 320, 321, 402, 403, 422, 437, 457, 458, 459, 460, 461, 462, 463, 464	50	96.40	0.75
